# Inhibition of Mitochondrial Redox Signaling with MitoQ Prevents Metastasis of Human Pancreatic Cancer in Mice

**DOI:** 10.3390/cancers14194918

**Published:** 2022-10-07

**Authors:** Tania Capeloa, Justine A. Van de Velde, Donatienne d’Hose, Sara G. Lipari, Françoise Derouane, Loïc Hamelin, Marie Bedin, Thibaut Vazeille, François P. Duhoux, Michael P. Murphy, Paolo E. Porporato, Bernard Gallez, Pierre Sonveaux

**Affiliations:** 1 Pole of Pharmacology and Therapeutics, Institut de Recherche Expérimentale et Clinique (IREC), Université Catholique de Louvain (UCLouvain), 1200 Brussels, Belgium; 2 Biomedical Magnetic Resonance Unit, Louvain Drug Research Institute (LDRI), Université Catholique de Louvain (UCLouvain), 1200 Brussels, Belgium; 3 Pole of Medical Imaging, Radiotherapy and Oncology, Institut de Recherche Expérimentale et Clinique (IREC), Université Catholique de Louvain (UCLouvain), 1200 Brussels, Belgium; 4 Department of Medical Oncology, Institut Roi Albert II, Cliniques Universitaires Saint-Luc, 1200 Brussels, Belgium; 5 MRC Mitochondrial Biology Unit, University of Cambridge, Cambridge CB2 0XY, UK; 6 Department of Molecular Biotechnology and Health Science, Molecular Biotechnology Center, University of Turin, 10126 Turin, Italy; 7 Walloon Excellence in Life Sciences and Biotechnology (WELBIO) Research Institute, 1300 Wavre, Belgium

**Keywords:** pancreatic ductal adenocarcinoma (PDAC), cancer metastasis, cancer metabolism, mitochondria, reactive oxygen species (ROS), redox signaling, MitoQ

## Abstract

**Simple Summary:**

If improvements of diagnostic techniques and methodologies allow to increasingly detect cancers at the premetastatic stage, metastases still account for about 90% of cancer patient deaths in the clinics. Indeed, there is currently no specific treatment capable of blocking or even delaying the metastatic process. In this context, we previously showed that mitochondria are metabolic sensors initiating the metastatic process. Here, focusing on pancreatic cancer, with which about one-third of patients are diagnosed before the onset of metastases, we report that MitoQ, a mitochondria-targeted antioxidant, interrupts prometastatic redox signaling from mitochondria to the cell body. At doses compatible with the treatment of humans, we found that MitoQ represses pancreatic cancer cell migration, invasion and clonogenicity. It reduced the metastatic homing of human pancreatic cancer cells in mice by about 50%. If applied to patients, metastasis prevention with MitoQ could increase the chances of curing cancer with conventional treatments.

**Abstract:**

At diagnosis, about 35% of pancreatic cancers are at the locally invasive yet premetastatic stage. Surgical resection is not a treatment option, leaving patients with a largely incurable disease that often evolves to the polymetastatic stage despite chemotherapeutic interventions. In this preclinical study, we hypothesized that pancreatic cancer metastasis can be prevented by inhibiting mitochondrial redox signaling with MitoQ, a mitochondria-targeted antioxidant. Using four different cancer cell lines, we report that, at clinically relevant concentrations (100–500 nM), MitoQ selectively repressed mesenchymal pancreatic cancer cell respiration, which involved the inhibition of the expression of PGC-1α, NRF1 and a reduced expression of electron-transfer-chain complexes I to III. MitoQ consequently decreased the mitochondrial membrane potential and mitochondrial superoxide production by these cells. Phenotypically, MitoQ further inhibited pancreatic cancer cell migration, invasion, clonogenicity and the expression of stem cell markers. It reduced by ~50% the metastatic homing of human MIA PaCa-2 cells in the lungs of mice. We further show that combination treatments with chemotherapy are conceivable. Collectively, this study indicates that the inhibition of mitochondrial redox signaling is a possible therapeutic option to inhibit the metastatic progression of pancreatic cancer.

## 1. Introduction

Although with a current incidence rate of 0.5% to 1% pancreatic ductal adenocarcinoma (PDAC) is still a rare cancer [1], it is projected to become the second leading cause of cancer-related mortality in the U.S. by 2030 [2]. At diagnosis, 10–20% of patients present a localized primary tumor, 30–35% present a locally advanced unresectable primary tumor and 50–55% have a metastatic disease [3]. For patients with locally advanced non-resectable or metastatic cancer, chemotherapy with gemcitabine alone or in combination with other chemotherapies prolongs life expectancy by a few months only, explaining why the overall patient survival rate at 5 years is ~10% only. Like for other cancer types, the emergence with time of treatment-resistant metastases is a main cause of death [4]. From a more optimistic point of view, these epidemiological numbers also indicate that about half of PDAC patients are diagnosed before the onset of metastases, which leaves a theoretical opportunity to apply treatment strategies aimed at preventing metastatic dissemination. However, to date, no such treatment exists in clinical practice, as no drug specifically targets and inactivates metastatic progenitor cells. At best, chemotherapies alone or in combination may retard the metastatic colonization of distant organs. They may further slowdown the growth or even eradicate established metastases, but they do not halt the metastatic process per se.

Preclinical work from our team [5,6,7] has demonstrated that metastatic dissemination can be prevented pharmacologically, even after cancers enter the metastatic stage. Based on the old observation that metastatic progenitor cells are selected in metabolically hostile areas in primary tumors (characterized, e.g., by hypoxia, nutrient deprivation, and acidosis) [8], our initial hypothesis was that these cells must possess metabolic sensors and transduction systems triggering escape. In mouse melanoma and breast cancer models, we identified the mitochondrion as a key metabolic sensor producing mitochondrial reactive oxygen species (mtROS) under nutritional stress conditions, with mitochondrial superoxide (mtO_2_^●−^) generation acting as a redox messenger to activate cancer cell migration, invasion and metastasis [5,6,7]. That a subcytotoxic increase in mtO_2_^●−^ levels is sufficient to trigger the metastatic process as a whole was demonstrated by Ishikawa et al. [9] who showed that transferring mtO_2_^●−^-producing mitochondria isolated from metastatic progenitor cells to nonmetastatic cells also transferred the metastatic phenotype. Upstream of mtO_2_^●−^, several mitochondrial events (including expressional defects of electron transport chain [ETC] subunits and ETC overload) were reported to increase electron leak, mtO_2_^●−^ production and metastasis [5]. Downstream, several mtO_2_^●−^-sensitive signaling pathways may relay the prometastatic information, among which we previously identified focal adhesion kinase (FAK) PYK2 in the transforming growth factor β (TGFβ) pathway as a prometastatic effector of mtO_2_^●−^ [5]. For therapeutic applications with limited compensatory/resistance mechanisms, one should therefore consider to directly target mitochondrial redox signaling.

Direct intervention to decrease mitochondrial oxidative stress has recently been made possible by the synthesis of mitochondria-targeted antioxidants, among which mitoquinol mesylate (MitoQ, wherein antioxidant ubiquinone is linked to a triphenylphosphonium [TPP^+^] group responsible for the mitochondrial accumulation of MitoQ driven by the negative mitochondrial membrane potential [ΔΨ]) [10], is an attractive therapeutic candidate [11]. Compared to general antioxidants that have unpredictable effects on primary tumor development as they notably interfere with anticancer immunity [12], MitoQ specifically targets mitochondrial redox signaling. 

In this study, we aimed to preclinically test the capacity of MitoQ to prevent PDAC metastasis. Using four different human PDAC cell lines, we report that MitoQ selectively acts on those producing detectable amounts of mtO_2_^●−^. At clinically relevant doses of 100–500 nM [6,11], MitoQ indeed dose-dependently interfered with PDAC cell respiration, resulting in decreased mtO_2_^●−^ production and signaling via proliferator-activated receptor γ coactivator-1α (PGC-1α) and nuclear respiratory factor 1 (NRF1). Phenotypically, MitoQ inhibited PDAC cell migration, invasion, clonogenicity and metastasis in mice, which should trigger the future evaluation of this drug in PDAC patients. In our view, if confirmed clinically, retarding or even blocking metastatic dissemination with MitoQ would provide more time and treatment opportunities to oncologists to try to cure the primary tumor.

## 2. Materials and Methods

### 2.1. Chemicals and Reagents

Mitoquinol mesylate (MitoQ) was produced as previously described [10], and dissolved in DMSO at a stock concentration of 10 mM. Solutions of 5-fluorouracil (5-FU; 50 mg/mL) and gemcitabine (38 mg/mL) were kindly provided by the Central Pharmacy of the Cliniques universitaires Saint-Luc (CUSL), Brussels, Belgium. Unless stated otherwise, all other chemicals were from Sigma-Aldrich (Overijse, Belgium). Equal volumes of solvent (DMSO) were used in control experiments.

### 2.2. Cells and Cell Culture

PANC1 (CRL-1469), MIA PaCa-2 (CRL-1420), Capan-1 (HTB-79) and HPAF-II (CRL-1997) human PDAC cell lines were from the American Tissue Cell Culture (ATCC, Manassas, VA, USA). PANC1 [13] and MIA PaCa-2 [14] cancer cells were originally derived from primary tumors of patients with invasive PDACs, HPAF-II from the ascites of a patient with metastatic PDAC [15], and Capan-1 from a liver metastasis of a patient with metastatic PDAC [16].

PANC1 and MIA PaCa-2 cancer cells were routinely cultured in DMEM containing 4.5 g/L glucose and GlutaMax (ThermoFisher, Erembodegem, Belgium; catalogue #10566016) with 10% FBS, HPAF-II in MEM alpha containing 4.5 g/L glucose and GlutaMax (ThermoFisher; catalogue #32571-028) with 10% FBS, and Capan-1 in IMDM containing GlutaMax (ThermoFisher; catalogue #31980030) with 20% FBS. Cells were maintained at a subconfluent state in a humidified atmosphere with 95% O_2_ and 5% CO_2_, 37 °C. Cell authenticities were confirmed with short tandem repeat (STR) tests (Eurofins Genomics, Ebersberg, Germany) (Table S1).

To constitutively express luciferase and the green fluorescent protein (GFP), MIA PaCa-2 cells were infected with lentiviral particles carrying the luciferase and GFP sequences along with a puromycin resistance gene (Amsbio, Alkmaar, The Netherlands; catalogue #LPV020). Briefly, 70–80% confluent cells were transduced using lentiviral particles (multiplicity of infection = 1) in 1 mL of DMEM supplemented with 10% FBS and 10 µL/mL polybrene. Cells were selected by a 48-72 h incubation with 2 µg/mL puromycin (InvivoGen, Kampenhout, Belgium), and FACS-sorted for GFP expression on a Becton Dickinson FACSAria III sorter (Erembodegem, Belgium). 

### 2.3. Metabolic Assays

Oxygen consumption rates (OCRs) and extracellular acidification rates (ECARs) were determined on a Seahorse XF96 bioenergetic analyzer using the XF Cell Mito Stress kit (Agilent Technologies, Machelen, Belgium), as previously reported [7] and detailed in Appendix A.1. 

Glucose and lactate concentrations were measured in cell supernatants collected after 48 h of culture ± MitoQ, using specific enzymatic assays on a CMA600 analyzer (Aurora Borealis, Schoonebeek, The Netherlands) [17]. All data were normalized by total protein content (Protein Assay from Bio-Rad, Temse, Belgium). 

Mitochondrial superoxide levels were determined using electron paramagnetic resonance (EPR) on a Bruker EMX-Plus spectrometer (Kontich, Belgium) operating in X-band (9.85 GHz) and equipped with a PremiumX ultra-low-noise microwave bridge (Bruker) and a SHQ high sensitivity resonator (Bruker), as previously reported [7,18] and detailed in Appendix A.2.

### 2.4. Immunocytochemistry

Cells treated ± MitoQ were cultured on glass coverslips, fixed in 4% paraformaldehyde (PFA), permeabilized with 0.1% Triton X-100 in PBS containing 0.1% Tween 20, and blocked with 5% BSA. Immunofluorescent staining was then performed as previously described [19]. Primary antibodies are listed in Table S2. Secondary antibodies were an Alexa Fluor 488-conjugated goat anti-rabbit (ThermoFisher; catalogue #A-11034) and an Alexa Fluor 488-conjugated goat anti-mouse (ThermoFisher; catalogue #A-28175). Nuclei were stained with 4′,6-diamidino-2-phenylindole dihydrochloride (DAPI, 1 µg/mL, Sigma-Aldrich), and F-actin using phalloidin-650 (ThermoFisher; 1/250). Images were captured by structured illumination fluorescence microscopy using an ApoTome-equipped AxioImager.z1 microscope (Zeiss, Zaventem, Belgium). Fluorescence intensity analyses were performed using the ImageJ software (NIH, Bethesda, MD, USA). Mitochondrial network analyses were performed using the MiNa toolset on ImageJ [20].

### 2.5. Western Blotting

Western blotting was performed as previously reported [7]. Procedures are detailed in Appendix A.3. Primary antibodies are listed in Table S2.

### 2.6. Mitochondrial Turnover Rate

The cellular mitochondrial turnover rate was measured using the pMitoTimer reporter gene (Addgene, Watertown, MA, USA; catalogue #52659), in which the pDsRed2-Mito sequence encoding the mitochondria-targeting sequence of human cytochrome c oxidase subunit VIII is fused to mutant DsRed1-E5 [21]. Mutant DsRed1-E5, which constitutes the timer, fluoresces like GFP when newly synthesized, and irreversibly shifts to red fluorescence upon maturation [22]. Therefore, cells with a high mitochondrial turnover rate essentially produce green fluorescence, whereas those with a low mitochondrial turnover rate produce more red fluorescence. Briefly, 300,000 cells were seeded on a cover slip, allowed to attach overnight, and were then transfected with 5 µg of pMitoTimer following manufacturer’s indications, using the Invitrogen Lipofectamine Transfection Reagent (ThermoFisher; catalogue #L3000-015) in Optimem (ThermoFisher; catalogue #31985062). After 24 h of transfection, medium was replaced by DMEM containing 4.5 g/L glucose and GlutaMax with 10% FBS, and the cells were treated ± MitoQ. After 48 h of treatment, the cells were washed with PBS, fixed with PFA 4% for 10 min, stained with DAPI for 30 min, rinsed 3 times with PBS, and imaged using a LSM800 confocal microscope (Zeiss). Images were analyzed using ImageJ, and the data were used to calculate red/green ratios. 

### 2.7. Real-Time Quantitative PCR

RT-qPCR was performed using a previously disclosed protocol [7] on a ViiA 7 Real-Time PCR System (ThermoFisher). Primers are listed in Table S3. All data were normalized to *β-actin* gene expression.

### 2.8. Mitochondrial Potential

The mitochondrial potential (ΔΨ) was measured using the JC-10 Mitochondrial Membrane Potential Assay Kit from Abcam (Cambridge, UK; catalogue #ab112134) following manufacturer’s recommendations. Briefly, 10,000 cells/well in 96-well plates were treated for 48 h ± MitoQ. After treatment, cells were washed twice with PBS, and stained with JC-10 (1x solution) for 45 min. Fluorescence intensities were read at 490/525 nm and 540/525 nm of absorbance using a SpectraMax i3 spectrophotometer equipped with a MiniMax imaging cytometer (Molecular Devices, Munich, Germany).

### 2.9. Cell Viability

For cell viability assays, 2500–5000 cells/well in 96-well plates were treated with increasing concentrations of MitoQ in their respective normal culture media for different durations (24 h, 48 h, 72 h). The same procedure was followed to test the effects of combination treatments with gemcitabine (0.05–20 μM) or 5-FU (5–320 µg/mL) ± MitoQ for 48 h and 72 h. After the end of treatments, the medium was discarded, and cells were then fixed in 4% PFA and stained with crystal violet (0.23% *v/v*). The dye retained by the cells was solubilized in 10% acetic acid, and the optical density (570 nm) was measured using a SpectraMax i3 spectrophotometer equipped with a MiniMax imaging cytometer. 

### 2.10. Cell Death

Cells were seeded in a 24-multiwell plate (10,000 cells per well) and allowed to adhere overnight. They were then treated with increasing doses of MitoQ and cultured for 72 h. Apoptosis and necrosis were determined using the Annexin V Apoptosis Detection kit FITC (ThermoFisher; catalogue #88-8005-74) according to manufacturer’s recommendations. Profiles were determined by FACS on a Canto II flow cytometer (BD Biosciences). A minimum of 5000 events were acquired for each sample.

### 2.11. Mouse Experiments

All in vivo interventions were performed on anesthetized (2% isoflurane, 98% oxygen) 5-week-old female Rj:NMRI-Foxn1 nu/nu mice (Janvier, Le Genest-Saint-Isle, France). 

For orthotopic tumor inoculation, 10^6^ MIA PaCa-2 cells constitutively expressing luciferase and GFP were embedded in 50 µL of matrix (25 µL of DMEM containing 4.5 g/L glucose and GlutaMax with 10% FBS + 25 µL of Matrigel [Sigma-Aldrich; catalogue #CLS354234]) and surgically injected into the tail of the pancreas according to the procedure of Chai M et al. [23]. Metastatic development was monitored once a week using a Xenogen IVIS 50 bioluminescence imaging system (PerkinElmer, Mechelen, Belgium). For image acquisition, mice were injected i.p. with 0.15 mg/g bodyweight of luciferin (PerkinElmer) and anesthetized using isoflurane after a 10-min incubation time. Bioluminescence was quantified with the Living Image software (PerkinElmer).

For tumor take assays, 100,000 MIA PaCa-2 cells constitutively expressing luciferase in 100 µL of PBS were injected in the left ventricle of mouse heart, using a 26G hypodermic needle and ultrasound guidance (Vevo 3100; Fujifilm VisualSonics, Amsterdam, The Netherlands). 

Mice were sacrificed by cervical dislocation under terminal anesthesia 5 weeks (orthotopic injection) or 10 weeks (metastatic take assays) after tumor cell inoculation. Chemiluminescence was acquired on organs ex vivo using the Xenogen IVIS 50 bioluminescence imaging system. Tumors and tissues were further processed for RNA and protein extraction. For immunohistochemistry, the collected lungs were embedded in paraffin, sectioned at 5 µm width, and 6 slices per mouse lung were stained with hematoxylin/eosin (H&E). Images of whole lung slices were acquired on a SCN400 Slide Scanner (Leica Biosystems, Diegem, Belgium) and analyzed with the Cytomine Software (Cytomine Corporation SA, Liège, Belgium; accessed on 10 December 2021). Quantification was performed according to Chang and Erler [24], and the number of lung metastases was normalized to the sliced lung area.

### 2.12. Cell Migration and Invasion Assays

Cell migration was evaluated using scratch assay and cell invasion in transwells, as previously shown [7]. Procedures are detailed in Appendix A.4.

### 2.13. Clonogenic Assays

Soft agar colony-formation assays were performed using 500 cells per test, as previously described [7]. After 2 weeks, colonies were counted using an Axiovert 40 CFL microscope equipped with a MRC camera. 

### 2.14. Cell Cycle 

To evaluate the cell cycle, cancer cells at about 70% of confluence were synchronized overnight in DMEM (for PANC1 and MIA PaCa-2), EMEM (for HPAF-II) or IMDM (for Capan-1) supplemented with 0.1% FBS, and then cultivated in the corresponding complete medium with 10% FBS for a time equivalent to their doubling time. After synchronization, cells were treated ± MitoQ for 48 h, trypsinized, centrifuged at 500 G, and washed twice with PBS. They were fixed by adding 700 µL of ice-cold ethanol 100% in 300 µL of cell suspension in PBS. Cells were then washed twice with 1 mL of Tris buffer with 0.2% (*v/v*) Triton X-100, and finally resuspended in 300 µL of PBS with RNase (0.2 mg/mL) and propidium iodide (5 µg/mL). At least 20,000 to 30,000 events were recorded using a BD FACSCalibur flow cytometer (Becton Dickinson). The FlowJo software v10.8 (Becton Dickinson) was used to process the data.

### 2.15. Statistics

All results are expressed as means ± standard error of the mean (SEM) for *n* independent observations. Error bars are sometimes smaller than symbols. Outliers were identified using Dixon’s Q test. Data were analyzed using Graphpad Prism version 9.2.0. (San Diego, CA, USA). Student’s *t*-test, one-way ANOVA with Dunnett’s post-hoc test and two-way ANOVA with Tukey’s post-hoc test were used where indicated. *p* < 0.05 was considered to be statistically significant.

## 3. Results

### 3.1. MitoQ Inhibits Human PDAC Cancer Cell Respiration

The aim of this study was to test preclinically whether inhibiting mitochondrial redox signaling with MitoQ has the potential to prevent metastatic progression in PDAC. We selected four human PDAC cell lines as working models, which, being based from a metabolic standpoint on Seahorse measurements in basal culture conditions, could be categorized as highly glycolytic/poorly oxidative (PANC1 and MIA PaCa-2) versus highly oxidative/poorly glycolytic (Capan-1 and HPAF-II) (Figure S1).

**Figure 1:**
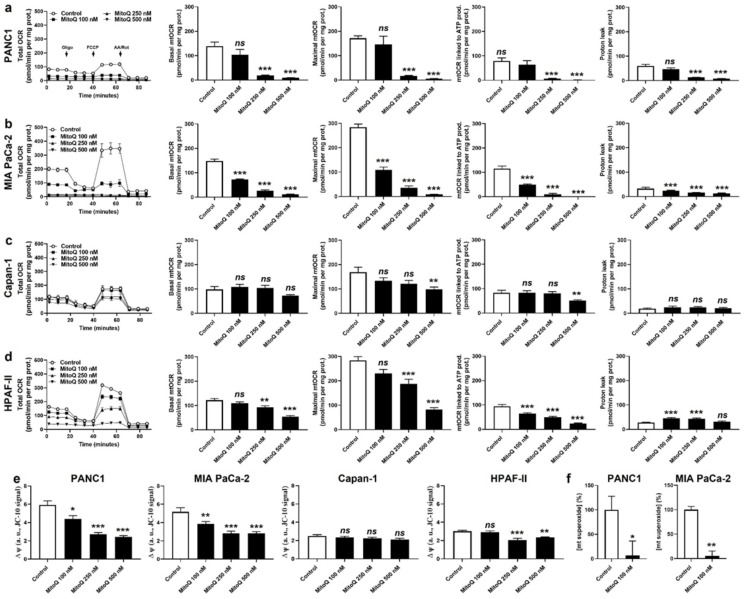
MitoQ represses mitochondrial respiration in human pancreatic cancer cells. (**a**–**f**) Cells were treated ± MitoQ for 48 h. (**a**) The oxygen consumption rate (OCR) of PANC1 cells was measured using Seahorse oximetry. The graph represents total OCR measurements over time with the sequential addition of oligomycin, FCCP, and rotenone (Rot) together with antimycin A (AA). From Seahorse traces, basal, maximal and ATP-linked mitochondrial OCRs (mtOCRs) and proton leak were calculated (*n* = 11–12). (**b**) Seahorse oximetry as in (a), but using MIA PaCa-2 cells (*n* =15–18). (**c**) Seahorse oximetry as in (a) but using Capan-1 cells (*n* = 9–12). (**d**) Seahorse oximetry as in (a), but using HPAF-II cells (*n* = 15–18). (**e**) The mitochondrial potential (∆ψ) was measured using JC-10 in PANC1, MIA PaCa-2, Capan-1 and HPAF-II cells (*n* = 12–18). (**f**) Mitochondrial superoxide (mtO_2_^●−^) levels were measured using electron paramagnetic resonance (EPR) with MitoTEMPO-H as a selective mtO_2_^●−^ sensor ± PEG-SOD2 in PANC1 (left; *n* = 9) and MIA PaCa-2 (right; *n* = 3) cells treated ± MitoQ 100 nM. All data are shown as means ± SEM. * *p* < 0.05, ** *p* < 0.01, *** *p* < 0.005, ns: *p* > 0.05, compared to control; by one-way ANOVA followed by Dunnett’s post hoc test (**a**–**e**) or Student’s *t*-test (**f**).

In vitro, MitoQ used at clinically relevant concentrations dose-dependently reduced the mitochondrial oxygen consumption rates (mtOCRs) of PANC1 and MIA PaCa-2 cells, as well as proton leak (Figure 1a,b). At 500 nM, which represents a concentration that can be achieved in tissues following a single dose delivery [6,25], MitoQ almost completely blocked basal mtOCR, maximal mtOCR and mtOCR linked to ATP production in the two cell lines. Comparatively, MitoQ had almost no effect on the mtOCR of Capan-1 cells (Figure 1c), and reduced the mtOCR of HPAF-II cells, albeit to a lesser extent compared to PANC1 and MIA PaCa-2 cells (Figure 1d). The mitochondrial proton leak of HPAF-II cells was slightly yet significantly increased at intermediate doses of MitoQ (100–250 nM), but not at the highest tested dose of 500 nM (Figure 1d).

As a logical consequence of OXPHOS inhibition [26], MitoQ dose-dependently decreased ΔΨ in PANC1 and MIA PaCa-2 cells (Figure 1e). No effects were seen in Capan-1 cells, and HPAF-II cells had an intermediate sensitivity to MitoQ (Figure 1e). MitoQ further decreased basal mtO_2_^●−^ levels in PANC1 and MIA PaCa-2 cells (Figure 1f), which was established using EPR with mitoTEMPO-H as a specific mitochondrial superoxide probe and PEG-SOD2 to specifically assign signals to mtO_2_^●−^ [18]. Capan-1 and HPAF-II cells did not produce detectable levels of mtO_2_^●−^.

**Figure 2:**
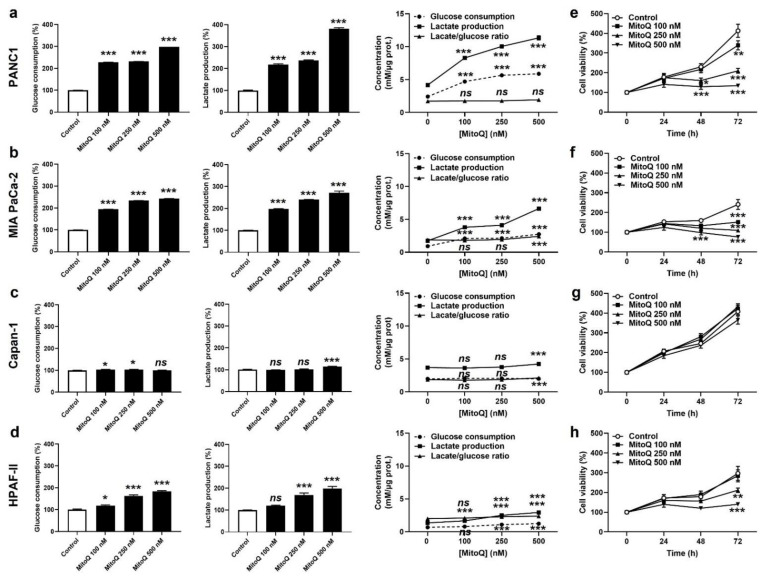
MitoQ is cytostatic for human pancreatic cancer cells. (**a**) PANC1 cells were treated ± MitoQ for 48 h. Glucose consumption (left), lactate production (middle) and the lactate/glucose ratio (right) were then determined using enzymatic assays on a CMA600 enzymatic analyzer (*n* = 3). (**b**) Enzymatic measurements of glucose consumption and lactate release as in (**a**), but using MIA PaCa-2 cells (*n* = 3). (**c**) Enzymatic measurements of glucose consumption and lactate release as in (**a**), but using Capan-1 cells (*n* = 3). (**d**) Enzymatic measurements of glucose consumption and lactate release as in (**a**), but using HPAF-II cells (*n* = 3). (**e**) PANC1 cell viability was determined using crystal violet staining (*n* = 12). (**f**) MIA PaCa-2 cell viability was determined as in (**e**) (*n* = 12). (**g**) Capan-1 cell viability was determined as in (**e**) (*n* = 11–12). (**h**) HPAF-II cell viability was determined as in (**e**) (*n* = 12). All data are shown as means ± SEM. * *p* < 0.05, ** *p* < 0.01, *** *p* < 0.005, ns: *p* > 0.05, compared to control; by one-way ANOVA followed by Dunnett’s post hoc test (**a**–**d**) or two-way ANOVA with Tukey’s post hoc test (**e**–**h**).

Cancer cells have a high metabolic plasticity. When experiencing OXPHOS inhibition, a common adaptation is that they increase their glycolytic rate (conversion of glucose to lactate) in order to replenish energy stores. Accordingly, PANC1 and MIA PaCa-2 cells exposed to MitoQ dose-dependently increased glucose consumption and lactate production rates, still keeping a steady-state glycolytic stoichiometry of two molecules of lactate released per molecule of glucose consumed (Figure 2a,b). Capan-1 cells barely adapted their glycolytic behavior to MitoQ (Figure 2c), and HPAF-II cells developed an intermediate response compared to PANC1 and MIA PaCa-2 (Figure 2d). Metabolic compensation by PANC1 and MIA PaCa-2 was associated with cell survival despite a dose-dependent decrease in cell proliferation (Figure 2e,f). Indeed, MitoQ was cytostatic at low doses, and only the highest dose of 500 nM of MitoQ turned out to be cytotoxic for MIA PaCa-2 cells, as it induced apoptosis but no necrosis (Figure S2). Comparatively, Capan-1 cell proliferation was not affected by MitoQ (Figure 2g), and HPAF-II cells were more sensitive to the cytotoxic effects of MitoQ than MIA PaCa-2 (Figure 2h), with doses of MitoQ ≥ 250 nM inducing HPAF-II cell apoptosis (Figure S2).

### 3.2. In Human PDAC Cancer cells, MitoQ Decreases the Expression of Nuclear Respiratory Factor 1 and Complexes I to III of the Electron Transport Chain

Compared to Coenzyme Q10 (CoQ10) that transfers electrons from ETC Complexes I and II to Complex III, MitoQ behaves as a chain-breaking antioxidant: it captures electrons at Complexes I and II, but is unable to donate these electrons to Complex III for steric reasons [27]. Mild ETC uncoupling may explain how MitoQ reduced OXPHOS in our model cancer cell lines, but because MitoQ also directly reacts with free radicals, we envisioned additional possible redox mechanisms.

We first focused on PGC-1α, a main contributor to mitochondrial biogenesis known to be mtROS-inducible [28]. Redox regulation occurs at transcriptional and posttranscriptional levels, with (mt)ROS triggering PGC-1α gene and protein expression and stabilizing the protein [29]. Conversely, MitoQ reduced PGC-1α protein expression in PANC1, MIA PaCa-2 and HPAF-II cells (Figure S3a). PGC-1α protein expression did not change in Capan-1 cells, which were globally poorly sensitive to MitoQ in all experiments. Downstream, MitoQ did not affect (MIA PaCa-2, HPAF-II) or slightly increased (PANC1, Capan-1) the intracellular mitochondrial density, as determined using immunofluorescence-based Tom20 staining and quantification (Figure S3b–e). Overall, MitoQ did not modify the cellular mitochondrial turnover rate (globally comprising fission, mitophagy, mitochondrial biogenesis and fusion) (Figure S3f), which was determined using MitoTimer [21]. We concluded that inhibition of PGC-1α-dependent mitochondrial biogenesis is not a main cause of OXPHOS inhibition by MitoQ.

**Figure 3:**
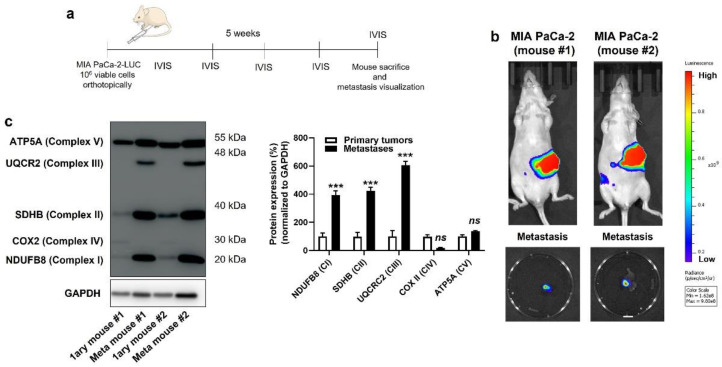
Compared to their respective primary tumors, human pancreatic cancer metastases in mice have an increased expression of key components of mitochondrial electron transport chain Complexes I, II and III. (**a**) Experimental protocol for spontaneous metastasis in NMRI nude mice bearing orthotopic MIA PaCa-2 human pancreatic tumors. (**b**) On the top are representative IVIS images of two mice bearing bioluminescent pancreatic primary tumors with constitutive luciferase expression. Images on the bottom show ex vivo bioluminescence signals of peritoneal lymph node metastases from the same 2 mice (bar = 500 µm). A scale bar is provided (arbitrary units). (**c**) Primary tumors and lymph node metastases from 2 independent MIA PaCa-2-bearing mice were microdissected and analyzed for protein expression in whole lysates. On the left, a representative Western blot shows the expression of NUDFB8, SDHB, UQCR2, COX2 and ATP5A, key components of mitochondrial electron transport chain Complex I (CI), Complex II (CII), Complex III (CIII), Complex VI (CIV), and Complex V (CV), respectively. GAPDH expression was used as a loading control. Data are quantified in the graph on the right (*n* = 4 all). *** *p* < 0.005, ns: *p* > 0.05, compared to control; by Student’s *t*-test (**c**).

Based on the above observations, we decided to broaden the scope of our investigation by comparing the OXPHOS machinery between primary human PDAC tumors and their own metastases. In living mice, PDAC cells were injected in the tail of the pancreas following the protocol of Chai M et al. [23] (Figure 3a and Figure S4a), which involved the production of cell lines constitutively expressing GFP and luciferase, laparotomy, pancreas exteriorization and postsurgical bioluminescence monitoring. All four cell lines were tested, but only MIA PaCa-2 generated primary orthotopic tumors that were metastatic in NMRI nude mice. Five weeks after implantation, primary tumors and lymph node metastases were detected using bioluminescence imaging (Figure 3b). After terminal anesthesia, several samples (to take into account tumor heterogeneity) were collected from each mouse, and lysates were processed for Western blotting, using a total OXPHOS human-antibody cocktail targeting ETC-complex subunits known to be labile when the complexes are not assembled [30]. We observed increased expression of ETC Complexes I, II and III, but not IV and V, in lymph-node metastases compared to primary MIA PaCa-2 tumors (Figure 3c). Ethical endpoints were reached before the onset of distant metastases, which prevented a similar comparison for distant colonization sites.

**Figure 4:**
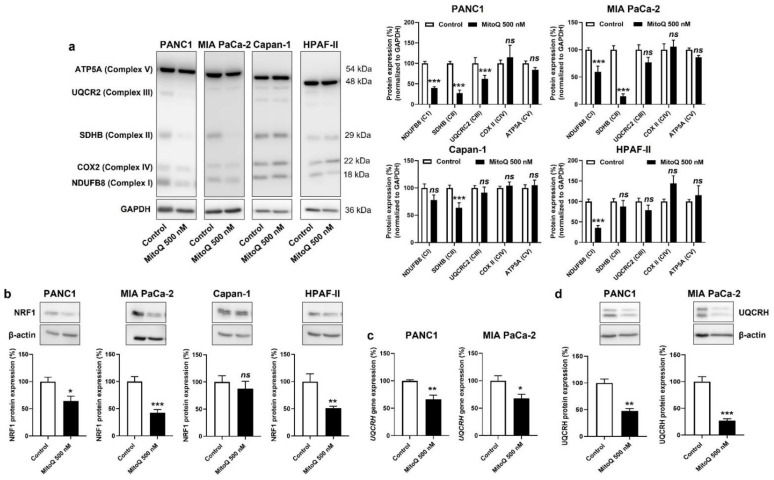
Inhibition of mitochondrial redox signaling by MitoQ decreases NRF1 expression and the expression of electron transport chain complexes in human pancreatic cancer cells. (**a**–**d**) PANC1, MIA PaCa-2, Capan-1 and HPAF-II cells were treated ± MitoQ for 48 h. (**a**) On the left, representative Western blots show the expression of NUDFB8, SDHB, UQCR2, COX2 and ATP5A, key components of mitochondrial electron transport chain Complex I (CI), Complex II (CII), Complex III (CIII), Complex VI (CIV), and Complex V (CV), respectively. GAPDH expression was used as a loading control. Data are quantified in the graphs on the right (*n* = 6–9). (**b**) Representative Western blots and graphs showing NRF1 protein expression in the cancer cells (*n* = 7 all). β-actin expression was used as a loading control. (**c**) UQCRH gene expression determined using RT-qPCR in PANC1 (left; *n* = 5–6) and MIA PaCa-2 (right; *n* = 4) cells. (**d**) Representative Western blots and graphs show UQCRH protein expression in the cancer cells (*n* = 4 all). β-actin expression was used as a loading control. All data are shown as means ± SEM. * *p* < 0.05, ** *p* < 0.01, *** *p* < 0.005, ns: *p* > 0.05, compared to control; by Student’s *t*-test (**a**–**d**).

In vitro, MitoQ reduced the expression of ETC Complexes I, II and III in PANC1; I and II in MIA PaCa-2; II in Capan-1; and I in HPAF-II cells (Figure 4a). Despite some variability in the responses of the different PDAC cell lines, only Complexes I to III were affected, whereas the expression of Complexes IV and V was insensitive to MitoQ. As expected, Capan-1 was the less sensitive cell line.

Upstream, NRF1 is a master inducer of the expression of nuclear encoded genes of mitochondrial respiratory complex subunits [31,32], and NRF1 activity has previously been reported to promote cancer metastasis [33]. If on the one hand mtROS-inducible PGC-1α coactivates NRF1 [34], on the other hand mtROS further induce NRF1 gene and protein expression [35,36]. Conversely, we found that MitoQ inhibits NRF1 protein expression in PANC1, MIA PaCa-2 and HPAF-II, but not in Capan-1 cells (Figure 4b). Using UQCRH gene and protein expression as a reporter of PGC-1α and NRF1 activities [37], we confirmed NRF1 repression by MitoQ (Figure 4c,d). A reanalysis of the tumor samples of Figure 3 using RT-qPCR and Western blotting further showed that UQCRH is enriched in lymph node metastases compared to primary MIA-PaCa-2 tumors (Figure S4a). Collectively, this series of experiments demonstrated that inhibition of the PGC-1α – NRF1 axis participates in OXPHOS inhibition by MitoQ in human PDAC cells.

### 3.3. Inhibition of Mitochondrial Redox Signaling Partially Reverses EMT in PDAC Cancer Cells

In the second part of the study, we analyzed the functional consequences of the inhibition of mitochondrial redox signaling by MitoQ on PDAC metastasis. 

**Figure 5:**
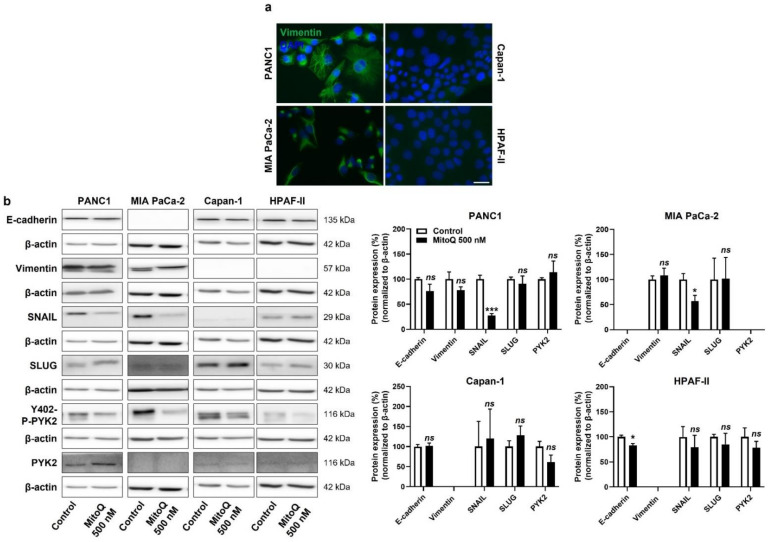
Inhibition of mitochondrial redox signaling partially represses EMT and the expression of EMT markers in human pancreatic cancer cells. (**a**) Representative immunocytofluorescence pictures show vimentin (green) and cell nuclei (DAPI, blue) staining in PANC1, MIA PaCa-2, Capan-1 and HPAF-II cell lines. Bar = 20 µm. (**b**) Cells were treated ± 500 nM of MitoQ for 48 h. Representative Western blots show the expression of EMT markers, as well as PYK2 and Y402-phospho-PYK2 (Y402-P-PYK2) in PANC1 (*n* = 3–5), MIA PaCa-2 (*n* = 4–5), Capan-1 (*n* = 5) and HPAF-II (*n* = 5) cells, and their expression is quantified on the graphs on the right. β-actin expression was used as a loading control. * *p* < 0.05, *** *p* < 0.005, ns: *p* > 0.05, compared to control; by Student’s *t*-test (**b**).

At the onset of metastasis, a partial epithelial-to-mesenchymal transition (EMT) precedes dissemination and the acquisition of stem cell properties [38]. Interestingly, similar to the categorization based on metabolic activities (Figure S1), our PDAC model cell lines could be categorized as more mesenchymal (PANC1, MIA PaCa-2) and more epithelial (Capan-1, HPAF-II) based on vimentin and E-cadherin expression (Figure 5a and Figure S5a,b). PANC1 and MIAPaCa-2 cells expressed higher levels of vimentin than Capan-1 and HPAF-II cells, and the opposite was observed for E-cadherin expression (high in Capan-1 and HPAF-II and low in PANC1 and MIAPaCa-2 cells) (Figure S5a). At the protein level, among the EMT markers that we analyzed, SNAIL expression was decreased upon cell exposure to MitoQ (Figure 5b). This response was seen in PANC1 and MIA PaCa-2 cells, whereas Capan-1 barely expressed SNAIL, and HDAF-II cells had lower, MitoQ-insensitive SNAIL expression compared to PANC1 and MIA PaCa-2. MitoQ slightly but significantly decreased E-cadherin expression in HPAF-II cells (Figure 5b).

For cell motion, we previously identified focal adhesion kinase PYK2, a main downstream effector of the TGF-β pathway [39], as a key effector of mtO_2_^●−^-dependent migration in B16 mouse melanoma models [5]. In PANC1 and Capan-1 cells, MitoQ inhibited PYK2 Y402-phosphorylation/activity (Figure 5b and Figure S5c), thus supporting the existence of a mtO_2_^●−^ – PYK2 pathway in some PDAC cancer cells. PYK2 was not expressed in MIA PaCa-2 cells, and there was no significant decrease in PYK2 Y402-phosphorylation/activity in HPAF-II cells treated with MitoQ (Figure 5b and Figure S5c).

### 3.4. Inhibition of Mitochondrial Redox Signaling Represses PDAC Cancer Cell Migration, Invasion and Clonogenicity

In addition to mesenchymal traits, metastatic progenitor cells must possess migration, invasion, clonogenic and stem-cell characteristics. In the context of metastasis prevention, we therefore tested whether inhibition of mitochondrial redox signaling by MitoQ could inhibit these phenotypes.

**Figure 6:**
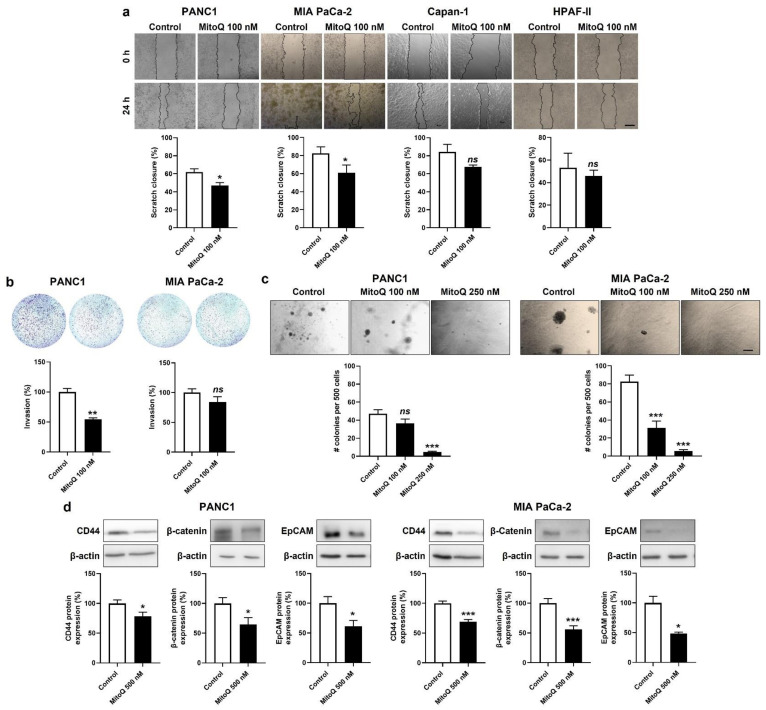
Inhibition of mitochondrial redox signaling represses migration, invasion, clonogenicity and the expression of stem cell markers by mesenchymal human pancreatic cancer cells. (**a**,**b**) Cells were treated ± 500 nM of MitoQ for 48 h. (**a**) PANC1 (*n* = 5), MIA PaCa-2 (*n* = 6), Capan-1 (*n* = 3) and HPAF-II (*n* = 3) cancer cell migration over 24 h was determined using a scratch assay. Representative pictures are shown on top and quantification graphs on the bottom. Bar = 50 µm. (**b**) PANC1 (*n* = 3) and MIA PaCa-2 (*n* = 7–8) cancer cell invasion was assayed in transwells with 1% FBS as chemoattractant. Representative images are shown together with overnight invasion data. Bar = 50 µm. (**c**) PANC1 (*n* = 16) and MIA PaCa-2 (*n* = 16) cancer cells were treated ± MitoQ for 20 days, followed by a clonogenic assay on soft agar. Representative images are shown together with quantification graphs. Bar = 50 µm. (**d**) Representative Western blots and graphs show the expression of stem cell markers CD44, β-catenin and epithelial cell adhesion molecule (EpCAM) in PANC1 (*n* = 16) and MIA PaCa-2 (*n* = 16) cancer cell treated ± 500 nM of MitoQ for 48 h. β-actin expression was used as a loading control. All data are shown as means ± SEM. * *p* < 0.05, ** *p* < 0.01, *** *p* < 0.005, ns: *p* > 0.05, compared to control; by Student’s *t*-test (**a**,**b**,**d**) or one-way ANOVA followed by Dunnett’s post hoc test (**c**).

Migration was tested in a scratch assay [7], where 100 nM of MitoQ significantly inhibited PANC1 and MIA PaCa-2, but not Capan-1 and HPAF-II cell migration (Figure 6a). At this stage, we put Capan-1 and HPAF-II cell lines aside, because of their global poor sensitivity to MitoQ.

Invasion was tested in transwell assays with 1% FBS as a chemoattractant and Matrigel mimicking the extracellular matrix. MitoQ (100 nM) inhibited PANC1, but not MIA Paca-2 cell invasion (Figure 6b). 

Clonogenicity was assayed on soft agar, where MitoQ dose-dependently inhibited PANC1 and MIA PaCa-2 colony formation (Figure 6c). A dose of 250 nM of MitoQ almost completely repressed the clonogenicity of both cell lines.

Stemness in PDAC is associated with a high expression of CD44, β-catenin and EpCAM [40]. PANC1 and MIA PaCa-2 expressed these three proteins in basal culture conditions, and inhibition of mitochondrial redox signaling by MitoQ significantly decreased their expression in the two cell lines (Figure 6d).

### 3.5. Inhibition of Mitochondrial Redox Signaling Inhibits Human PDAC Metastatic Homing in Mice

**Figure 7:**
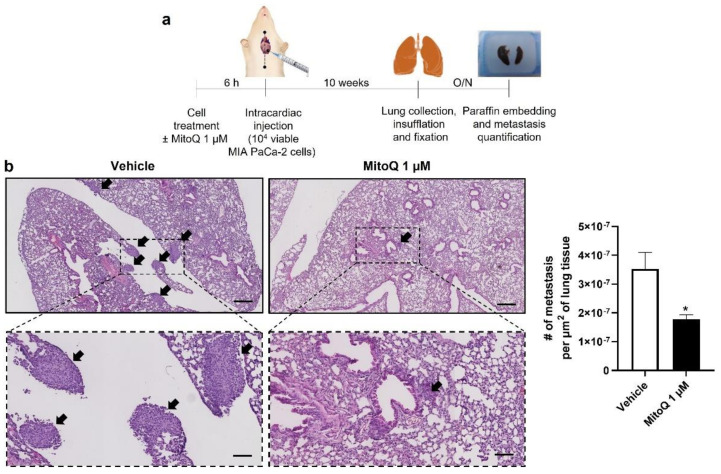
Inhibition of mitochondrial redox signaling by MitoQ inhibits the homing of circulating pancreatic cancer cells to mouse lungs. (**a**) Experimental protocol for MIA PaCa-2 metastasis to NMRI nude mouse lungs. (**b**) At the end of the protocol depicted in (**a**), mouse lungs were collected, sliced, stained with hematoxylin and eosin (H&E), and analyzed for the presence of metastases. Representative pictures are on the left at two different magnifications (bars = 400 µm for pictures on the top and 100 µm for insets), where metastases are indicated by black arrows. Quantification of the number of metastases per surface area is shown on the right (*n* = 4 mice in each condition). All data are shown as means ± SEM. * *p* < 0.05 compared to control, by Student’s *t*-test (**b**).

Homing and distant organ colonization are the final steps of the metastatic process, which can be tested in metastatic take assays in mice. Cancer cells were pretreated ±1 µM of MitoQ (a dose chosen taking into account dilution and clearance in the blood stream, as previously performed [5,6]) for 6 h, which was followed by an intracardiac injection of 10,000 viable cells as depicted in Figure 7a. Ten weeks later, untreated MIA PaCa-2 cells produced detectable lung metastases (Figure 7b). In this model, MitoQ reduced metastatic homing by half, which was quantified in six non-consecutive H&E-stained slices per mouse lung using a slide scanner. Similar to what we had seen with the orthotopic model (Figure 3a), PANC1 cells were non metastatic in NMRI nude mice. 

In the clinics, gemcitabine and 5-FU are two main antimetabolite drugs used to treat PDAC patients [3]. These drugs have been reported to trigger mtROS production [41], which could be responsible, at least in part, for their cytotoxic action on cancer cells. With translational motivations, we therefore concluded our study by testing the compatibility of combined treatments. In vitro, a direct cell count revealed that 100 nM of MitoQ did not influence PANC1, MIA PaCa-2, Capan-1 or HPAF-II cancer cell killing by gemcitabine (Figure S6a,b) nor by 5-FU (Figure S6c,d). It is of note that increasing doses of chemotherapy were tested with two time points in order to take into account the dose distribution heterogeneities that would arise in tumors in vivo, and early as well as delayed cell death.

## 4. Discussion

Our study aimed to test preclinically whether mitochondrial redox signaling inhibition by MitoQ is capable to prevent PDAC metastasis. Using four human cell lines as models, we identified responders (PANC1 and MIA Paca-2), a partial responder (HPAF-II) and a non-responder (Capan-1) to the treatment. Compared to partial and non-responders, responders were characterized by a higher glycolytic/OXPHOS ratio, detectable mtO_2_^●−^ production, and partial engagement in the EMT process (with high vimentin expression). They further expressed PDAC stem cell markers and were clonogenic in vitro, which collectively are essential characteristics of metastatic progenitor cells. However, only MIA PaCa-2 cancer cells were tumorigenic and metastatic in NMRI nude mice, thus at best recapitulating the metastatic progenitor cell phenotype among all tested cell lines. Focusing on responders, we report that MitoQ inhibits PDAC cell respiration, ΔΨ, mtO_2_^●−^ production, migration, invasion, clonogenicity and metastatic take to mouse lungs. 

Mechanistically, MitoQ is distinguished from general antioxidants by its local action on mtROS, owing to compound accumulation within the negatively charged mitochondrial matrix, which is driven by the TPP^+^ group of MitoQ [42]. There, it can directly react with free radicals, and oxidized mitoquinone is recycled to reduced mitoquinol by reacting with mitochondrial ETC Complex II [11,42]. MitoQ thus acts as a catalyst that blocks mitochondrial redox signaling, sparing other nonmitochondrial redox systems. The present study evidences that MitoQ also acts as a chain-breaker agent that dose-dependently decreases ΔΨ, proton leak (which correlates with the respiration rate [43]), cancer cell respiration and, ultimately, mtO_2_^●−^ production, as the ETC coupling efficiency is progressively disrupted. These two activities of MitoQ, direct reaction and mild ETC uncoupling, participate with still unknown relative degrees in blocking redox signaling from mitochondria to the cancer cell body.

Metastatic progenitor cells are characterized by OXPHOS activities higher than those of other isogenic cancer cells, resulting in a higher ΔΨ and higher superoxide production [5,6,7,9]. Using an orthotopic model of PDAC, our study confirms that circulating cancer cells that reach proximal lymph nodes on their metastatic route are better equipped for OXPHOS, as, compared to cancer cells in primary tumors, they overexpress ETC Complexes I to III. The selectivity of MitoQ for these cells, compared to other cancer cells and host cells within solid tumors, would explain, at least in part, why MitoQ does not interfere with primary tumor growth in mice [5,6], and why systemic toxicity is limited to nausea and vomiting at the maximal tolerated oral dose (1 mg/kg) of MitoQ [11,44,45]. Plasma concentrations reached in humans (~50 nM after single dose delivery) coupled to tissue accumulation over time and dose [11] are within the range in which metastatic prevention was achieved in mice [6,7] (and this study).

Downstream of mtO_2_^●−^, H_2_O_2_ has a half-life long enough to transmit the prometastatic redox signal originating at the ETC, and there is no mitochondrial isoform of catalase. Among many redox-sensitive signals, we previously demonstrated in a mouse melanoma model that mtROS signaling in metastatic progenitor comprises the activation of the TGFβ pathway at the level of src kinase (Y416 phosphorylation) directly within mitochondria [5], thus bypassing the need of TGFβ to bind to the TGFβ receptor for pathway activation. Once activated, src sequentially activates SMAD4 and FAK PYK2 (Y402 phosphorylation) [5], which executes the prometastatic program by remodeling the cytoskeleton [39]. Here, we confirmed basal PYK2 Y402-phosphorylation/activity in three out of four PDAC cell lines producing mtO_2_^●−^ (Mia PaCa-2 cells did not express PYK2 at all), and we showed that MitoQ reduces this phosphorylation. We further showed that the PGC-1α – NRF1 axis, which is mtROS-inducible [35,36] and promotes mitochondrial biogenesis and the expression of most nuclear encoded ETC subunits [31,32,34], was repressed by MitoQ in these cell models. While mitochondrial turnover was unaffected, inhibition of this axis by MitoQ is likely to participate in the OXPHOS deficiency associated with decreased mtO_2_ ^−^ levels that we documented. NRF1 activation also promotes EMT and produces cancer stem cells, thus facilitating the metastatic process [46]. Whether the decreased expression of EMT and stem cell markers that we observed in MitoQ-treated TNBC [7] and PDAC (this study) cells directly or indirectly results from NRF1 inhibition certainly warrants further investigation.

Phenotypically, MitoQ inhibited human PDAC cell migration, invasion, clonogenicity and metastatic homing in mice, with some differences between PANC1 and MIA PaCa-2 cells. Comparing two different cancer cells lines is indeed tricky, as their basal characteristics differ, even if they both represent the same cancer type at a same stage. In migration assays, for example, intrinsic differences were evident, as PANC1 cells naturally closed about 60% of the scratch wound whereas MIA PaCa-2 cells closed about 80% in 24 h. MIA PaCa-2 were also more clonogenic than PANC1 cells, which may explain why MIA PaCa-2 generated tumors that were metastatic in the lifetime of mice, whereas PANC1 cells did not. Intrinsic differences may also explain different sensitivities to MitoQ. For instance, compared to PANC1 cells that were sensitive to MitoQ in invasion assays, MIA PaCa-2 cells were not, and did not express PYK2, which is a major effector of mtROS signaling though the downstream part of the TGFβ pathway [5]. Other, yet-uncharacterized differences between the two cell lines are likely to account for their different metastatic capabilities and different sensibilities to MitoQ in phenotypic assays.

Based on this preclinical study and on preclinical studies with TNBC [6,7], we believe that MitoQ is a promising drug candidate to prevent cancer metastasis in vivo. Despite limitations inherent to preclinical mouse assays, the observation that MitoQ prevents metastatic homing to the lungs following I.V. cancer cell injection is important in our opinion, as it indicates that, in an individual with cancer, MitoQ would still repress metastatic dissemination after metastatic progenitor cell shedding in the blood stream. While metastatic prevention was almost complete in some TNBC models [5,6,7], we find very encouraging the ~50% prevention of human PDAC metastatic take in the lungs of mice that was achieved in this study. For patients diagnosed at a premetastatic stage, preventing metastatic dissemination would provide more chances of cure, as it would provide more time for oncologists to focus on primary cancer treatment with curative intentions. For patients diagnosed with an already metastatic cancer, the inhibition of mitochondrial redox signaling with MitoQ would still reduce and/or retard metastatic dissemination, which would increase their quality of life and, perhaps, their life expectancy (palliative intention). A clinical translation of our findings would be facilitated by the fact that MitoQ is orally bioavailable and already passed safety Phase I clinical trials [11,44,45]. Phase II clinical trials in cancer can therefore be concretely planned, pending the identification of an appropriate treatment regimen (dose schedule) and, if possible, for financial reasons, earlier read-outs (biomarkers) than metastatic onset in patients. It is with this same long-term aim in mind that we verified in vitro that MitoQ does not interfere with the PDAC cell killing activities of gemcitabine and 5-FU.

## 5. Conclusions

Conclusively, our preclinical study provides unprecedented evidence that human PDAC metastasis can be prevented pharmacologically, by selectively targeting mitochondrial redox signaling with mitochondria-targeted antioxidant MitoQ. The fact that MitoQ already cleared Phase I clinical trials [11] and is currently under Phase II investigation for other pathologies than cancer [44,45] should facilitate the clinical translation of our preclinical findings.

## 6. Patent

T.C. and P.S. are inventors of patent application EP21175397.5 “Molecular signature for assessing the responsiveness of cancer to mitochondria-targeted antioxidants” related to MitoQ.

## Data Availability

All data are contained within the article and supplementary material.

## Supplementary Materials

The following supporting information can be downloaded at: https://www.mdpi.com/article/10.3390/cancers14194918/s1, Figure S1: MitoQ does not interfere with the cell cycle of human pancreatic cancer cells; Figure S2: Only high doses of MitoQ induce the death of human pancreatic cancer cells. Figure S3: Inhibition of mitochondrial redox signaling by MitoQ decreases PGC-1α protein expression without decreasing mitochondrial density in human pancreatic cancer cell lines; Figure S4: Compared to primary orthotopic tumors in mice, lymph node metastases have increased UQCRH gene expression; Figure S5: MitoQ decreases vimentin expression in human pancreatic cancer cell lines; Figure S6: In vitro, inhibition of mitochondrial redox signaling does not interfere with conventional chemotherapies used to treat pancreatic cancer; Figure S7: Uncropped Western blot images; Table S1: Short tandem repeat profiles; Table S2: Antibodies used for Western blotting and immunofluorescence; Table S3: Primers used in quantitative PCR.

## Appendix A

### Appendix A.1. Seahorse Assays

For Seahorse assays, 10,000 cells/well were plated on XF96 culture plates (Agilent Technologies) 16 h before experiments in DMEM containing 4.5 g/L glucose and GlutaMax (ThermoFisher; catalogue #10566016) and 10% FBS, and treated ± MitoQ for 48 h. On the day of analysis, culture medium was replaced by DMEM containing 10 mM glucose, 2 mM glutamine, 1.85 g/L NaCl, 3 mg/L phenol red, pH 7.4. Cells were incubated for 1 h in a CO_2_-free incubator before analyses. Sequentially, basal OCR was acquired without treatment; ATP-linked OCR after the addition of 1 µM of ATP synthase inhibitor oligomycin; maximal OCR after mitochondrial potential disruption using 1 µM of ionophore carbonyl cyanide-4-(trifluoromethoxy)phenylhydrazone (FCCP); and non-mitochondrial OCR after the addition of 0.5 µM of Complex I inhibitor rotenone together with 0.5 µM of Complex III inhibitor antimycin A. mtOCRs were calculated by subtracting non-mitochondrial OCRs from corresponding basal, maximal and ATP-linked OCRs. Respiration used for ATP production is the difference between basal OCR and OCR in the presence of ATP-synthase inhibitor oligomycin. All data were normalized by total protein content.

### Appendix A.2. EPR Measurements of Mitochondrial Superoxide Levels

For EPR measurement of mtO_2_^●−^ levels, cells were treated ± MitoQ for 48 h, harvested and resuspended in PBS at 2 × 10^6^ cells/mL. MitoTEMPO-H was used as a specific mitochondrial superoxide probe ± pegylated superoxide dismutase 2 (PEG-SOD2) to specifically assign signals to mitochondrial superoxide [18]. Net superoxide production was calculated by subtracting the double integration values of the MitoTEMPO-H spectrum with PEG-SOD2 to the control value at timepoint 15 min.

### Appendix A.3. Western Blotting

For cells in culture, whole-protein extracts from PANC1, MIA PaCa-2, HPAF–II and Capan-1 cancer cells treated ± MitoQ for 48 h were prepared and processed as previously described [7]. For tissues from primary and secondary tumors, tumor samples collected immediately after mouse sacrifice were chopped and lysed using RIPA buffer (50 mM Tris pH 7.4, 150 mM NaCl, 1% Triton-X-100, 0.05 % sodium deoxycholate, 1 mM EDTA, 0.1% SDS, protease inhibitor cocktail and PhosSTOP Phosphatase Inhibitor Cocktail [Sigma-Aldrich]), and processed as the cell lysates [7]. β-actin or GAPDH served as a loading controls. PageRuler Prestained Protein Ladder (ThermoFisher; catalogue #26616) was used as molecular weight marker.

OXPHOS complexes were detected using the Total OXPHOS Human WB Antibody Cocktail mouse anti-ATP5A; UQCRC2; SDHB; COXII; NDUFB8 from Abcam (catalogue #ab110411; 1:1000). Immunodetection was performed at room temperature for 1 h using as secondary antibodies a horseradish peroxidase-conjugated goat anti-rabbit (The Jackson Laboratory, Sacramento, CA, USA; catalogue #111-035-003) and goat anti-mouse (The Jackson Laboratory; catalogue #115-035-003) in PBS-Tween containing 5% (*w*/*v*) milk or BSA. Detection was performed with ECL reagent (Amersham brand of GE Healthcare, Diegem, Belgium; catalogue #RPN2209), and protein bands were visualized and captured using ECL imager 600 (Amersham). They were analyzed using the ImageJ software.

Uncropped Western blot images are available in Figure S7.

### Appendix A.4. Cell Migration and Invasion Assays

For migration assays, cells were treated ± MitoQ for 48 h, and pictures were analyzed using ImageJ. The percentages of scratch closure were calculated with respect to the wounded area at time 0.

Cell invasion was tested in transwell assays on Corning Falcon 24-well plates (Avantor, Leuven, Belgium; catalogue # 62406-198). Briefly, the upper side of insert membranes (8-µM diameter pores) was coated with 250 µg/ml Matrigel (Sigma-Aldrich; catalogue #CLS354234), during 2 h at 37 °C. A total of 100,000 cells/well were then seeded in the upper compartment in serum-free DMEM containing 4.5 g/L glucose and GlutaMax (ThermoFisher; catalogue #10566016), while the same medium supplemented with 1% FBS was used as chemoattractant in the bottom chamber. After 16 h in routine culture conditions, the transwell was removed, and washed with PBS. Cells on the bottom side of membranes were wiped using a cotton swap, fixed in 4% PFA for 10 min and stained with crystal violet (0.23% *v/v*). Pictures were acquired on an Axiovert 40 CFL microscope (Zeiss) equipped with a MRC camera (Zeiss). Quantification was performed using the QuPath software v0.1.2 [47].

## References

[1] Siegel R.L., Miller K.D., Fuchs H.E., Jemal A. (2021). Cancer statistics, 2021. CA Cancer J. Clin..

[2] Rahib L., Smith B.D., Aizenberg R., Rosenzweig A.B., Fleshman J.M., Matrisian L.M. (2014). Projecting cancer incidence and deaths to 2030: The unexpected burden of thyroid, liver, and pancreas cancers in the United States. Cancer Res..

[3] Park W., Chawla A., O’Reilly E.M. (2021). Pancreatic cancer: A review. JAMA.

[4] Zeng S., Pottler M., Lan B., Grutzmann R., Pilarsky C., Yang H. (2019). Chemoresistance in pancreatic cancer. Int. J. Mol. Sci..

[5] Porporato P.E., Payen V.L., Perez-Escuredo J., De Saedeleer C.J., Danhier P., Copetti T., Dhup S., Tardy M., Vazeille T., Bouzin C. (2014). A mitochondrial switch promotes tumor metastasis. Cell Rep..

[6] Capeloa T., Krzystyniak J., Canas Rodriguez A., Payen V.L., Zampieri L.X., Pranzini E., Derouane F., Vazeille T., Bouzin C., Duhoux F.P. (2022). MitoQ prevents human breast cancer recurrence and lung metastasis in mice. Cancers.

[7] Capeloa T., Krzystyniak J., d’Hose D., Canas Rodriguez A., Payen V.L., Zampieri L.X., Van de Velde J.A., Benyahia Z., Pranzini E., Vazeille T. (2022). MitoQ inhibits human breast cancer cell migration, invasion and clonogenicity. Cancers.

[8] Schlappack O.K., Zimmermann A., Hill R.P. (1991). Glucose starvation and acidosis: Effect on experimental metastatic potential, DNA content and MTX resistance of murine tumour cells. Br. J. Cancer.

[9] Ishikawa K., Takenaga K., Akimoto M., Koshikawa N., Yamaguchi A., Imanishi H., Nakada K., Honma Y., Hayashi J. (2008). ROS-generating mitochondrial DNA mutations can regulate tumor cell metastasis. Science.

[10] Kelso G.F., Porteous C.M., Coulter C.V., Hughes G., Porteous W.K., Ledgerwood E.C., Smith R.A., Murphy M.P. (2001). Selective targeting of a redox-active ubiquinone to mitochondria within cells: Antioxidant and antiapoptotic properties. J. Biol. Chem..

[11] Smith R.A., Murphy M.P. (2010). Animal and human studies with the mitochondria-targeted antioxidant MitoQ. Ann. N. Y. Acad. Sci..

[12] Porporato P.E., Sonveaux P. (2015). Paving the way for therapeutic prevention of tumor metastasis with agents targeting mitochondrial superoxide. Mol. Cell. Oncol..

[13] Lieber M., Mazzetta J., Nelson-Rees W., Kaplan M., Todaro G. (1975). Establishment of a continuous tumor-cell line (PANC1) from a human carcinoma of the exocrine pancreas. Int. J. Cancer.

[14] Yunis A.A., Arimura G.K., Russin D.J. (1977). Human pancreatic carcinoma (MIA PaCa-2) in continuous culture: Sensitivity to asparaginase. Int. J. Cancer..

[15] Chauhan S.S., Shetty A.B., Hatami E., Chowdhury P., Yallapu M.M. (2020). Pectin-tannic acid nano-complexes promote the delivery and bioactivity of drugs in pancreatic cancer cells. Pharmaceutics.

[16] Fogh J., Fogh J.M., Orfeo T. (1977). One hundred and twenty-seven cultured human tumor cell lines producing tumors in nude mice. J. Natl. Cancer Inst..

[17] Sonveaux P., Vegran F., Schroeder T., Wergin M.C., Verrax J., Rabbani Z.N., De Saedeleer C.J., Kennedy K.M., Diepart C., Jordan B.F. (2008). Targeting lactate-fueled respiration selectively kills hypoxic tumor cells in mice. J. Clin. Investig..

[18] Scheinok S., Capeloa T., Porporato P.E., Sonveaux P., Gallez B. (2020). An EPR study using cyclic hydroxylamines to assess the level of mitochondrial ROS in superinvasive cancer cells. Cell. Biochem. Biophys..

[19] Zampieri L.X., Grasso D., Bouzin C., Brusa D., Rossignol R., Sonveaux P. (2020). Mitochondria participate in chemoresistance to cisplatin in human ovarian cancer cells. Mol. Cancer Res..

[20] Valente A.J., Maddalena L.A., Robb E.L., Moradi F., Stuart J.A. (2017). A simple ImageJ macro tool for analyzing mitochondrial network morphology in mammalian cell culture. Acta Histochem..

[21] Laker R.C., Xu P., Ryall K.A., Sujkowski A., Kenwood B.M., Chain K.H., Zhang M., Royal M.A., Hoehn K.L., Driscoll M. (2014). A novel MitoTimer reporter gene for mitochondrial content, structure, stress, and damage in vivo. J. Biol. Chem..

[22] Terskikh A., Fradkov A., Ermakova G., Zaraisky A., Tan P., Kajava A.V., Zhao X., Lukyanov S., Matz M., Kim S. (2000). “Fluorescent timer”: Protein that changes color with time. Science.

[23] Chai M.G., Kim-Fuchs C., Angst E., Sloan E.K. (2013). Bioluminescent orthotopic model of pancreatic cancer progression. J. Vis. Exp..

[24] Chang J., Erler J.T. (2016). Quantification of lung metastases from in vivo mouse models. Adv. Exp. Med. Biol..

[25] James A.M., Sharpley M.S., Manas A.R., Frerman F.E., Hirst J., Smith R.A., Murphy M.P. (2007). Interaction of the mitochondria-targeted antioxidant MitoQ with phospholipid bilayers and ubiquinone oxidoreductases. J. Biol. Chem..

[26] Muller F.L., Liu Y., Van Remmen H. (2004). Complex III releases superoxide to both sides of the inner mitochondrial membrane. J. Biol. Chem..

[27] Smith R.A., Hartley R.C., Cocheme H.M., Murphy M.P. (2012). Mitochondrial pharmacology. Trends Pharmacol. Sci..

[28] Rabinovitch R.C., Samborska B., Faubert B., Ma E.H., Gravel S.P., Andrzejewski S., Raissi T.C., Pause A., St-Pierre J., Jones R.G. (2017). AMPK maintains cellular metabolic homeostasis through regulation of mitochondrial reactive oxygen species. Cell Rep..

[29] Rius-Perez S., Torres-Cuevas I., Millan I., Ortega A.L., Perez S. (2020). PGC-1alpha, inflammation, and oxidative stress: An integrative view in metabolism. Oxid. Med. Cell Longev..

[30] Olahova M., Peter B., Szilagyi Z., Diaz-Maldonado H., Singh M., Sommerville E.W., Blakely E.L., Collier J.J., Hoberg E., Stranecky V. (2021). POLRMT mutations impair mitochondrial transcription causing neurological disease. Nat. Commun..

[31] van der Lee R., Szklarczyk R., Smeitink J., Smeets H.J., Huynen M.A., Vogel R. (2015). Transcriptome analysis of complex I-deficient patients reveals distinct expression programs for subunits and assembly factors of the oxidative phosphorylation system. BMC Genomics.

[32] Scarpulla R.C. (2008). Nuclear control of respiratory chain expression by nuclear respiratory factors and PGC-1-related coactivator. Ann. N. Y. Acad. Sci..

[33] Liu W., Beck B.H., Vaidya K.S., Nash K.T., Feeley K.P., Ballinger S.W., Pounds K.M., Denning W.L., Diers A.R., Landar A. (2014). Metastasis suppressor KISS1 seems to reverse the Warburg effect by enhancing mitochondrial biogenesis. Cancer Res..

[34] Wu Z., Puigserver P., Andersson U., Zhang C., Adelmant G., Mootha V., Troy A., Cinti S., Lowell B., Scarpulla R.C. (1999). Mechanisms controlling mitochondrial biogenesis and respiration through the thermogenic coactivator PGC-1. Cell.

[35] Miranda S., Foncea R., Guerrero J., Leighton F. (1999). Oxidative stress and upregulation of mitochondrial biogenesis genes in mitochondrial DNA-depleted HeLa cells. Biochem. Biophys. Res. Commun..

[36] Suliman H.B., Carraway M.S., Welty-Wolf K.E., Whorton A.R., Piantadosi C.A. (2003). Lipopolysaccharide stimulates mitochondrial biogenesis via activation of nuclear respiratory factor-1. J. Biol. Chem..

[37] Zaza G., Granata S., Masola V., Rugiu C., Fantin F., Gesualdo L., Schena F.P., Lupo A. (2013). Downregulation of nuclear-encoded genes of oxidative metabolism in dialyzed chronic kidney disease patients. PLoS ONE.

[38] Palamaris K., Felekouras E., Sakellariou S. (2021). Epithelial to mesenchymal transition: Key regulator of pancreatic ductal adenocarcinoma progression and chemoresistance. Cancers.

[39] Wendt M.K., Schiemann B.J., Parvani J.G., Lee Y.H., Kang Y., Schiemann W.P. (2013). TGF-beta stimulates Pyk2 expression as part of an epithelial-mesenchymal transition program required for metastatic outgrowth of breast cancer. Oncogene.

[40] Patil K., Khan F.B., Akhtar S., Ahmad A., Uddin S. (2021). The plasticity of pancreatic cancer stem cells: Implications in therapeutic resistance. Cancer Metastasis Rev..

[41] Fujiwara-Tani R., Sasaki T., Takagi T., Mori S., Kishi S., Nishiguchi Y., Ohmori H., Fujii K., Kuniyasu H. (2022). Gemcitabine resistance in pancreatic ductal carcinoma cell lines stems from reprogramming of energy metabolism. Int. J. Mol. Sci..

[42] Snow B.J., Rolfe F.L., Lockhart M.M., Frampton C.M., O’Sullivan J.D., Fung V., Smith R.A., Murphy M.P., Taylor K.M., Protect Study G. (2010). A double-blind, placebo-controlled study to assess the mitochondria-targeted antioxidant MitoQ as a disease-modifying therapy in Parkinson’s disease. Mov. Disord..

[43] Gane E.J., Weilert F., Orr D.W., Keogh G.F., Gibson M., Lockhart M.M., Frampton C.M., Taylor K.M., Smith R.A., Murphy M.P. (2010). The mitochondria-targeted anti-oxidant mitoquinone decreases liver damage in a phase II study of hepatitis C patients. Liver Int..

[44] Ross M.F., Prime T.A., Abakumova I., James A.M., Porteous C.M., Smith R.A., Murphy M.P. (2008). Rapid and extensive uptake and activation of hydrophobic triphenylphosphonium cations within cells. Biochem. J..

[45] Jastroch M., Divakaruni A.S., Mookerjee S., Treberg J.R., Brand M.D. (2010). Mitochondrial proton and electron leaks. Essays Biochem..

[46] Das J.K., Felty Q., Poppiti R., Jackson R.M., Roy D. (2018). Nuclear respiratory factor 1 acting as an oncoprotein drives estrogen-induced breast carcinogenesis. Cells.

[47] Bankhead P., Loughrey M.B., Fernandez J.A., Dombrowski Y., McArt D.G., Dunne P.D., McQuaid S., Gray R.T., Murray L.J., Coleman H.G. (2017). QuPath: Open source software for digital pathology image analysis. Sci. Rep..

