# Supplementary material for: Inhibition of Mitochondrial Redox Signaling with MitoQ Prevents Metastasis of Human Pancreatic Cancer in Mice
Source: Cancers (Basel). 2022 Oct 7;14(19):4918. doi: 10.3390/cancers14194918 (PMC9562676; doi:10.3390/cancers14194918)
Supplement: Supplementary file 1 [file cancers-14-04918-s001.zip › cancers-1936738-supplementary.pdf]

Inhibition of Mitochondrial Redox Signaling with MitoQ Prevents Metastasis of Human Pancreatic Cancer in Mice

Supplementary Tables

Table S1. Short tandem repeat profiles

	PANC1	MIA Paca-2	Capan-1	HPAF-II
**D8S1179**	16, 16	11, 12	14, 15	14, 16
**D21S11**	29, 31.2	31, 31	28, 28	28, 30
**D7S820**	12, 13	10, 13	8, 10	10, 11
**CSFF1PO**	10, 10	10, 11	10, 12	11, 11
**D3S1358**	16, 16	14, 18	17, 17	15, 15
**TH01**	9, 10	9, 9	7, 8	6, 6
**D13S317**	12, 13	12, 12	11, 11	9, 9
**D16S539**	10, 13	11, 13	11, 11	13, 14
**D2S1338**	25, 25	16, 19	23, 24	20, 24
**D19S433**	15, 15	12, 12	11, 16	14, 15
**vWA**	15, 15	17, 17	15, 15	16, 16
**TPOX**	9, 9	8, 8	8, 11	8, 11
**D18S51**	12, 12	13, 13	12, 12	12, 12
**AMEL**	X, X	X, X	X, X	X, X
**D5S818**	12, 13	11, 13	11, 13	11, 11
**FGA**	22, 22	21, 24	21, 21	24, 24

Table S2. Antibodies used for western blotting and immunofluorescence

Protein name	Antibody	Company & Catalogue #	Dilution
β-catenin	rabbit anti- β-catenin	Cell signaling, #9582P	1:1,000
CD44	rabbit anti-CD44	Abcam #Ab-51037	1:1,000
E-cadherin	rabbit anti-E-cadherin	Cell signaling, #3194	1:1,000
E-cadherin*	mouse anti-E-cadherin	Cell signaling, #14472S	1:50
EPCAM (CD326)	rabbit anti-EPCAM	Cell signaling, #14452, D9S3P	1:1,000
GAPDH	rabbit anti-GAPDH	Cell Signaling, #2118	1:1,000
NRF1	rabbit anti-NRF1	Cell signaling, #69432S	1:1,000
PGC-1α	Mouse anti- PGC-1α	Santa Cruz, #sc-518025	1:1,000
PYK2	rabbit anti-PYK2	Cell signaling, #3292S	1:1,000
P-PYK2	rabbit anti-Tyr402	Cell signaling, #3291S	1:1,000
SNAIL	rabbit anti-snail	Cell signaling, #3879	1:1,000
SLUG	rabbit anti-slug	Cell signaling, #9585	1:1,000
TWIST1	rabbit anti-twist1	Cell signaling, #46702	1:1,000
TOM20	rabbit anti-TOM20	Invitrogen, #PA5-52843	1:200
UQCRH	rabbit anti-UQCRH	Abcam, #ab134949	1:1,000
VIMENTIN	rabbit anti-vimentin	Cell signaling, #5741	1:1,000
VIMENTIN*	mouse anti-vimentin	Dako, #M0725	1:100
β-actin	mouse anti-β-actin	Sigma-Aldrich, #A5441	1:1,000

* Antibodies used for immunofluorescence.

Table S3. Primers used in quantitative PCR

Human gene name	Forward 5' - 3'	Reverse 5'- 3'
CYC1	CCAGATAGCCAAGGATGTG	ATCATCAACATCTTGAGCCC
MT-CYB	ATCACTTTATTGACTCCTAGCC	TGGTTGTCCTCCGATTCAG
NDUFA1	TTGGGTATCACTGGAGTCTG	TTCTCCAAACCCTTTGACAC
NDUFA5	TGCTGAAGAAGACCACTGG	CCAAAGTGCTGGGATTACAG
NDUFA10	GAGAGGAATTCATAGCAGTGTG	GCTTGAAGCCTAGTTTCTCTG
NDUFA12	GGAGTTAGTGCAGGTCCTG	TTCTTCGCATCATTTGTCCTG
UQCR10	*TCTACGACCACATCAACGA*	*CCACAGCTTCTCTACTCCA*
UQCR11	GGGTACCTTACATCAATGGC	AGTCTGTGAAGGGTTTGTG
UQCRB	GTGGACCAAATATGAAGAGGA	CCGAATAACCTCTTTCAGATACG
UQCRC1	*CTTTCAAGGGAACAAAGAATCG*	*CTGTAGGCATTAAGATGGGC*
UQCRFS1	*CATGTTGTCGGTAGCATCC*	*AGAAGCAGGGACATTGAGG*
UQCRQ	CACGTCTTCACTAAAGGAATCC	AAACACTACAAACTGCGGC
UQCRH	ATCTAGAACCGTAGCCAGAC	CCTCTTCCTCTTCCTCCTC
β-ACTIN	CCCGCGAGCACAGAGC	TCATCATCCATGGTGAGCTGG

Supplementary Figures

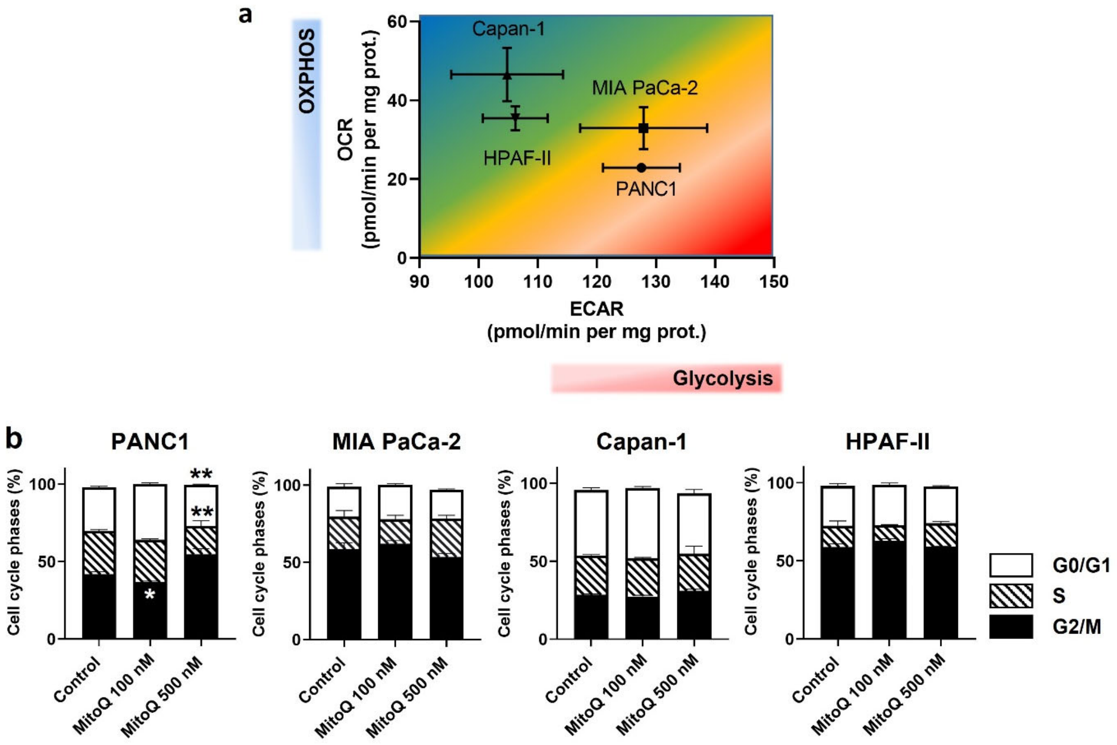

Figure S1. MitoQ does not interfere with the cell cycle of human pancreatic cancer cells. (**a**) The graph shows oxygen consumption rate (OCR, reflecting OXPHOS) on extracellular acidification rate (ECAR, reflecting glycolysis) plotted for PANC1 (n = 12), MIA PaCa-2 (n = 18), Capan-1 (n = 9) and HPAF-II (n = 15) cancer cells in complete medium. (**b**) PANC1, MIA PaCa-2, Capan-1 and HPAF-II cancer cells were treated ± MitoQ for 48 h. Cell cycle progression was determined using propidium iodide staining followed by flow cytometry. Graphs display the relative number of cells in each phase of the cell cycle (total = 100%). All data are shown as means ± SEM. * $p < 0.05$ compared to control; by 2-way ANOVA with Sidak's post hoc test (b).

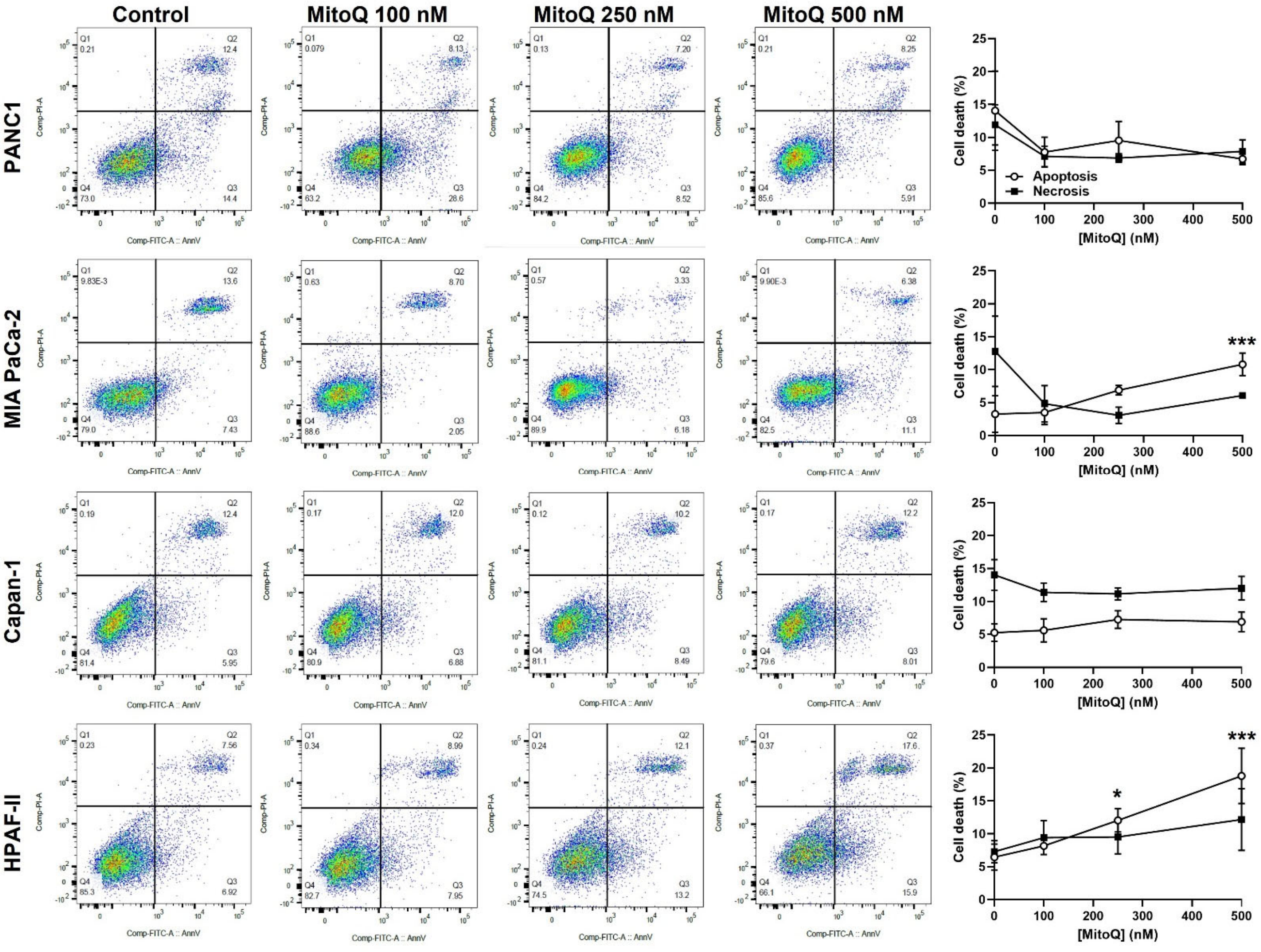

Figure S2. Only high doses of MitoQ induce the death of human pancreatic cancer cells. PANC1 (n = 3-4), MIA PaCa-2 (n = 3-4), Capan-1 (n = 4) and HPAF-II (n = 3-4) cancer cells were treated ± MitoQ for 72 h. Apoptosis and necrosis were determined using the Annexin V Apoptosis Detection kit FITC (ThermoFisher) and quantified on a Canto II flow cytometer. A minimum of 5000 events were acquired for each sample. * $p < 0.05$, *** $p < 0.005$; by one-way ANOVA with Dunnet's post hoc test.

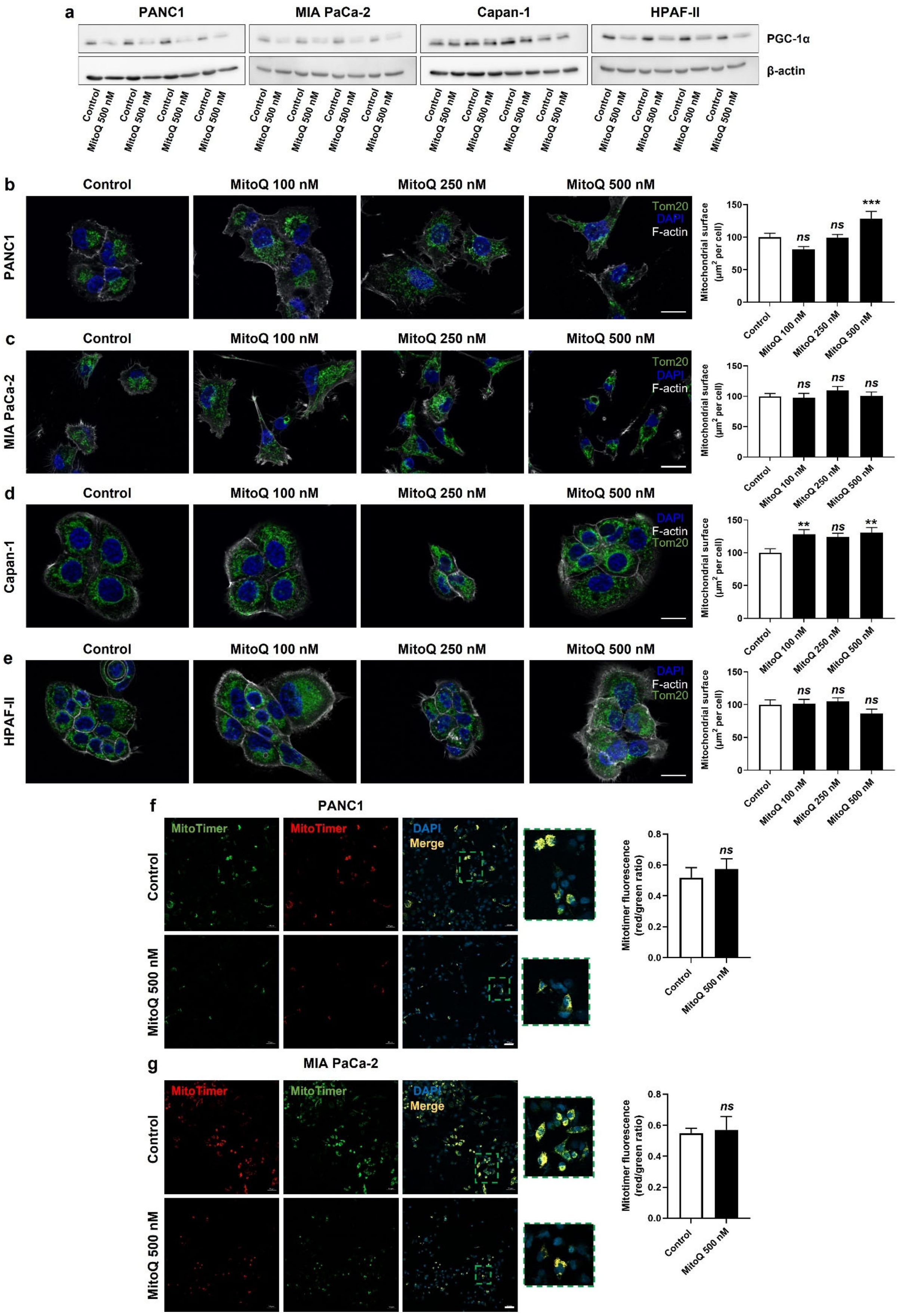

a
PANC1
MIA PaCa-2
Capan-1
HPAF-II
PGC-1α
β-actin
Control
MitoQ 500 nM
b
Control
MitoQ 100 nM
MitoQ 250 nM
MitoQ 500 nM
PANC1
Tom20
DAPI
F-actin
Mitochondrial surface (μm² per cell)
ns
***
c
MIA PaCa-2
d
Capan-1
**
e
HPAF-II
f
PANC1
MitoTimer
DAPI
Merge
Mitotimer fluorescence (red/green ratio)
g
MIA PaCa-2

Figure S3. Inhibition of mitochondrial redox signaling by MitoQ decreases PGC1-α protein expression without decreasing mitochondrial density in human pancreatic cancer cell lines. A-E, Cancer cells were treated ± MitoQ for 48 h. (**a**) Western blots show the protein expression of PGC1-α and β-actin, used as a loading control, in PANC1, MIA PaCA-2, Capan-1 and HPAF-II cells treated ± 500 nM MitoQ. (**b**) On the left, representative Immunofluorescence images show mitochondria labeled with an anti-Tom20 antibody (green), the cytoskeleton labeled with an anti-F-actin antibody (white) and cell nuclei stained with DAPI (blue). Bar = 20 μm. The left graph reports on mitochondrial density (*n* = 17-52). (**c**) Same as in (b) but using MIA PaCa-2 cells (*n* = 17-31). (**d**) Same as in (b) but using Capan-1 cells (*n* = 20-46). (**e**) Same as in (d) but using HPAF-II cells (*n* = 21-30). Representative pictures are shown on left. Graphs (right) represent the mitochondrial surface per cell (MitoFootprint). (**f**) The mitochondrial turnover rate in PANC1 cells treated for 48 h ± MitoQ 500nM was determined using MitoTimer. Representative images show green fluorescence (left, native reporter protein), red fluorescence (middle, mature reporter protein), and a merge (right). Bar = 50 μm. The right graph reports on the red/green fluorescence ratio (*n* = 4). (**g**) As in (f) but using MIA PaCa-2 cells (*n* = 4). All data are shown as means ± SEM. ** $p < 0.01$, *** $p < 0.005$, ns: $p > 0.05$ compared to control; by one-way ANOVA with Dunnett's post hoc test (**b-e**) or Student t test (**f, g**).

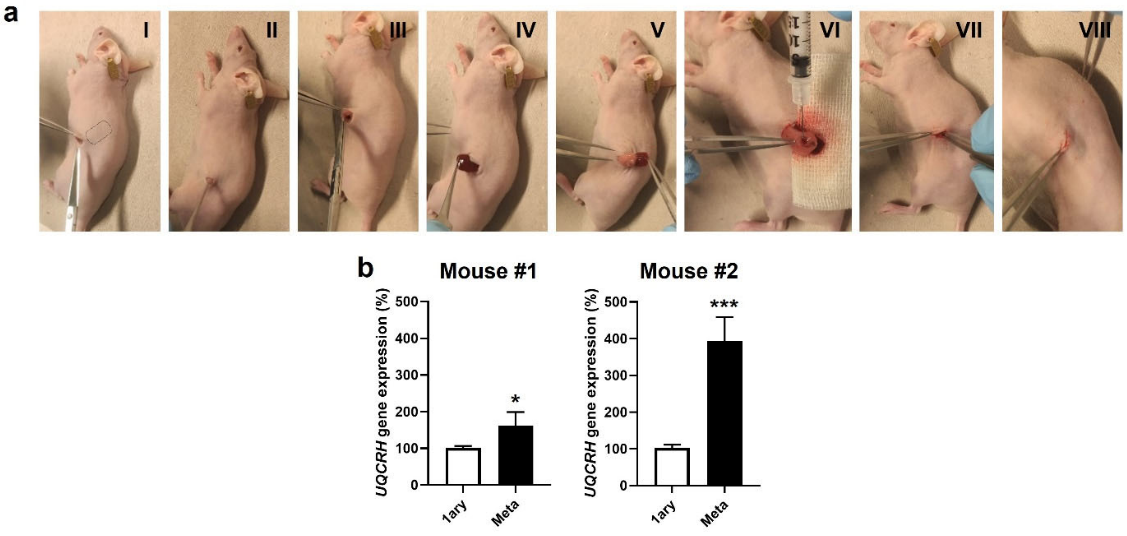

Figure S4. Compared to primary orthotopic tumors in mice, lymph node metastases have increased *UQCRH* gene expression. (**a**) Representative images illustrating the sequential steps of the surgical procedure used for orthotopic human pancreatic cancer cell injection in NMRI nude mice. (I) The abdomen of the anesthetized mouse is disinfected and a small skin incision is made under the spleen (this organ can be seen by the darker color under the mouse skin); (II) and (III) a small incision is made in the peritoneum; (IV) with the help of forceps, the spleen is gently exteriorized; (V) the pancreatic tail is exteriorized with the spleen and held in place; (VI) A sterile humid gauze is positioned under the spleen to keep the organs moistened during surgery, and Matrigel-embedded pancreatic cancer cells are gently injected into the pancreas tail; (VII) the organs are carefully placed inside the cavity of the abdomen; and (VIII) the wound is closed in two layers. (**b**) Two mice were treated as depicted in **Figure 3a**. The graphs show *UQCRH* gene expression detected using RT-qPCR in primary tumors and the corresponding lymph node metastases (*n* = 3-6 analyzed samples for mouse #1 and *n* = 5-6 for mouse #2). All data are shown as means ± SEM. * $p < 0.05$, *** $p < 0.005$ compared to control; by Student t test (**b**).

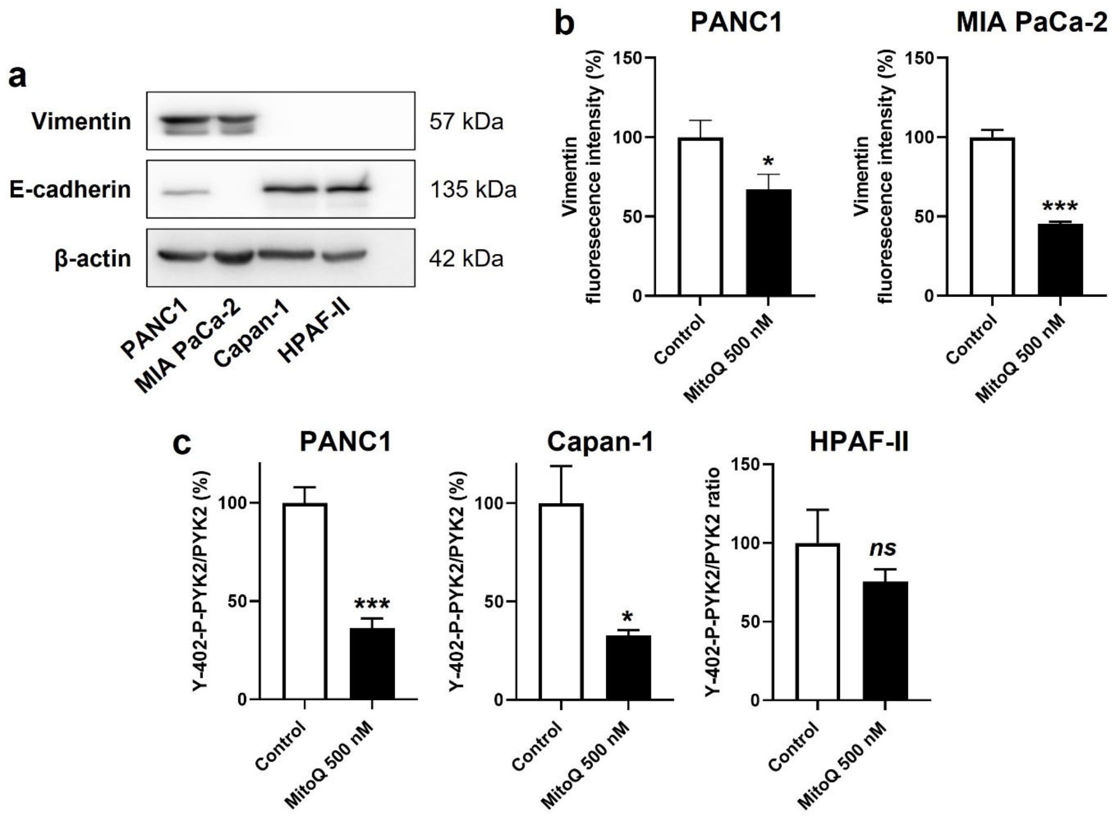

Figure S5. MitoQ decreases vimentin expression in human pancreatic cancer cell lines. (**a**) Representative western blots show the basal protein expression of vimentin, E-cadherin and β-actin, used as a loading control, in PANC1, MIA PaCA-2, Capan-1 and HPAF-II cells. (**b**) Cancer cells were treated ± 500 nM MitoQ for 48 h. The graphs show vimentin expression detected by immunocytofluorescence in PANC1 (*n* = 110-163) and MIA PaCa-2 (*n* = 275-338) cells, as illustrated in **Figure 5a**. (**c**) Cells were treated ± 500 nM of MitoQ for 48 h. The graphs show the ratio of Y402-phospho-PYK2 (Y402-P-PYK2) on total PYK2 detected by western blotting in PANC1 (*n* = 5), Capan-1 (*n* = 3-5) and HPAF-II (*n* = 5) cells, as illustrated in **Figure 5b**. All data are shown as means ± SEM. * $p < 0.05$, *** $p < 0.005$ compared to control; by Student t test (b, c).

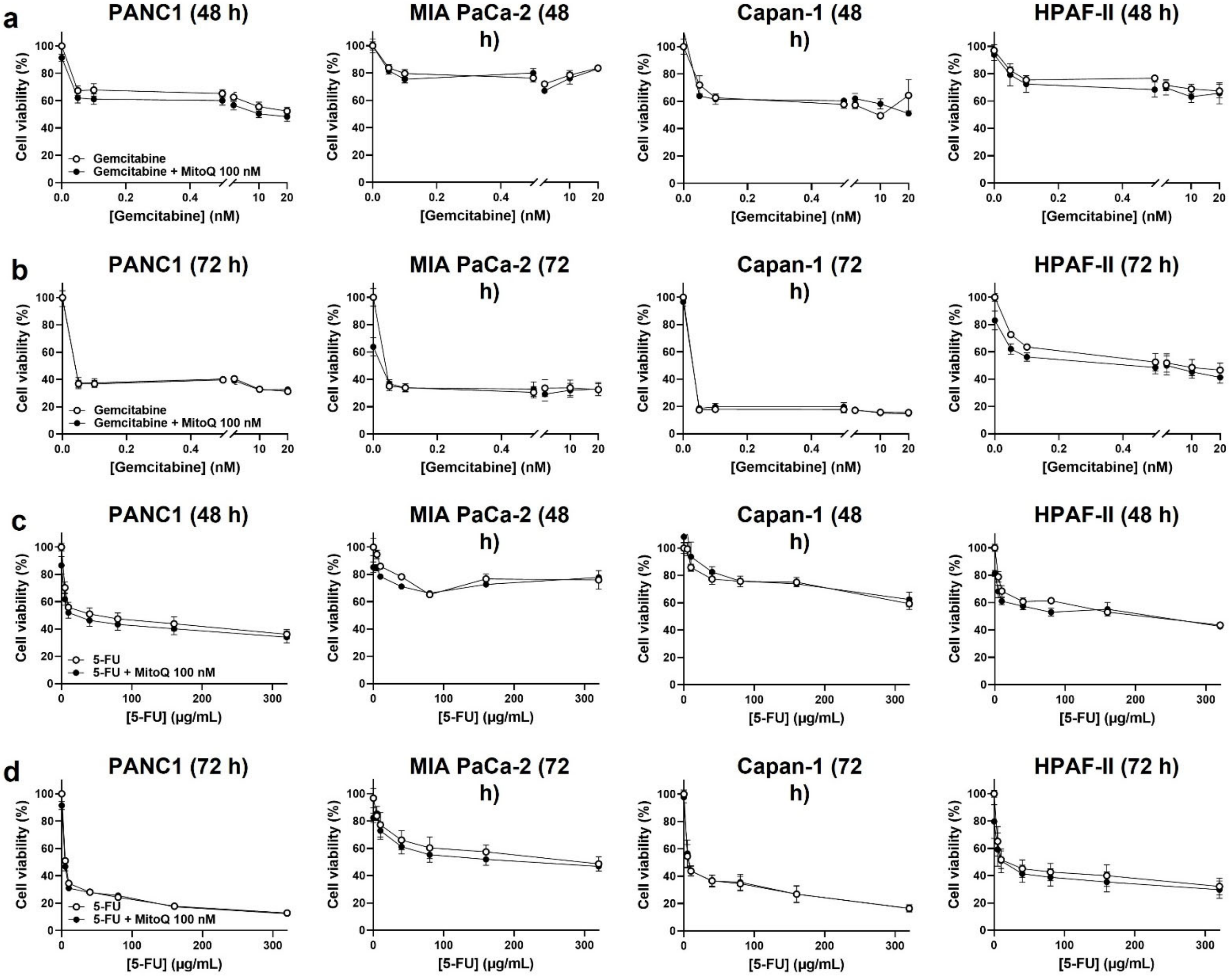

Figure S6. In vitro, inhibition of mitochondrial redox signaling does not interfere with conventional chemotherapies used to treat pancreatic cancer. (**a**) The viability of PANC1 (*n* = 8-9), MIA PaCA-2 (*n* = 3), Capan-1 (*n* = 3) and HPAF-II (*n* = 6) cells was determined using crystal violet staining after a treatment of 48 h with increasing doses of gemcitabine ± 100 nM MitoQ. (**b**) Viability of PANC1 (*n* = 6), MIA PaCA-2 (*n* = 6-7), Capan-1 (*n* = 7) and HPAF-II (*n* = 7) cells after a treatment of 72 h with increasing doses of gemcitabine ± 100 nM MitoQ. (**c**) Viability of PANC1 (*n* = 9), MIA PaCA-2 (*n* = 3), Capan-1 (*n* = 3) and HPAF-II (*n* = 3) cells after a treatment of 48 h with increasing doses of 5-fluorouracil (5-FU) ± 100 nM MitoQ. (**d**) Viability of PANC1 (*n* = 6), MIA PaCA-2 (*n* = 6-7), Capan-1 (*n* = 7) and HPAF-II (*n* = 7) cells after a treatment of 72 h with increasing doses of 5-FU ± 100 nM MitoQ. All data are shown as means ± SEM and were analyzed using two-way ANOVA.

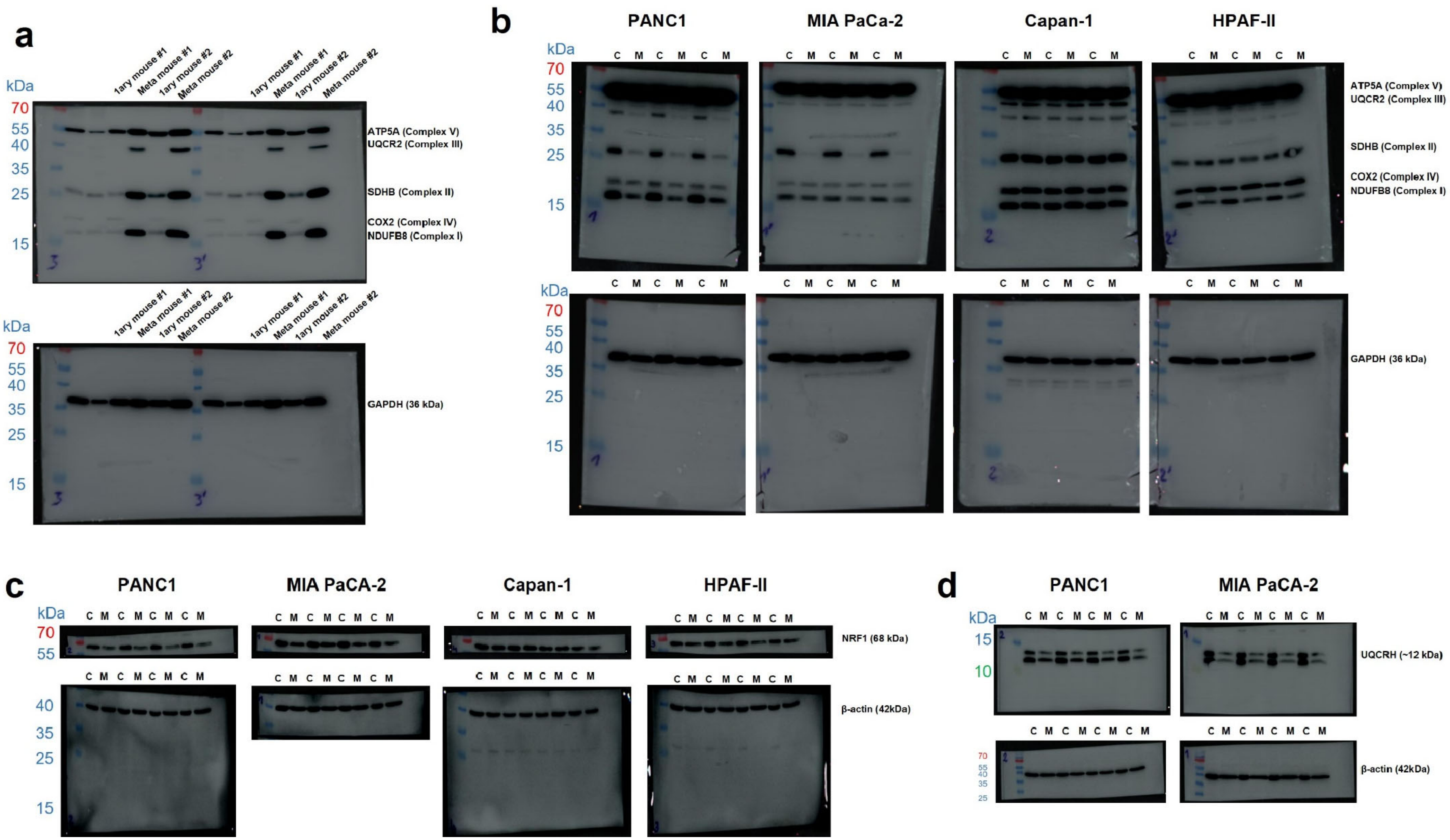
a
kDa
70
55
40
35
25
15
1ary mouse #1
Meta mouse #1
1ary mouse #2
Meta mouse #2
ATP5A (Complex V)
UQCR2 (Complex III)
SDHB (Complex II)
COX2 (Complex IV)
NDUFB8 (Complex I)
GAPDH (36 kDa)
b
PANC1
MIA PaCa-2
Capan-1
HPAF-II
C M C M C M
c
PANC1
MIA PaCA-2
Capan-1
HPAF-II
NRF1 (68 kDa)
β-actin (42kDa)
d
PANC1
MIA PaCA-2
10
UQCRH (~12 kDa)
β-actin (42kDa)

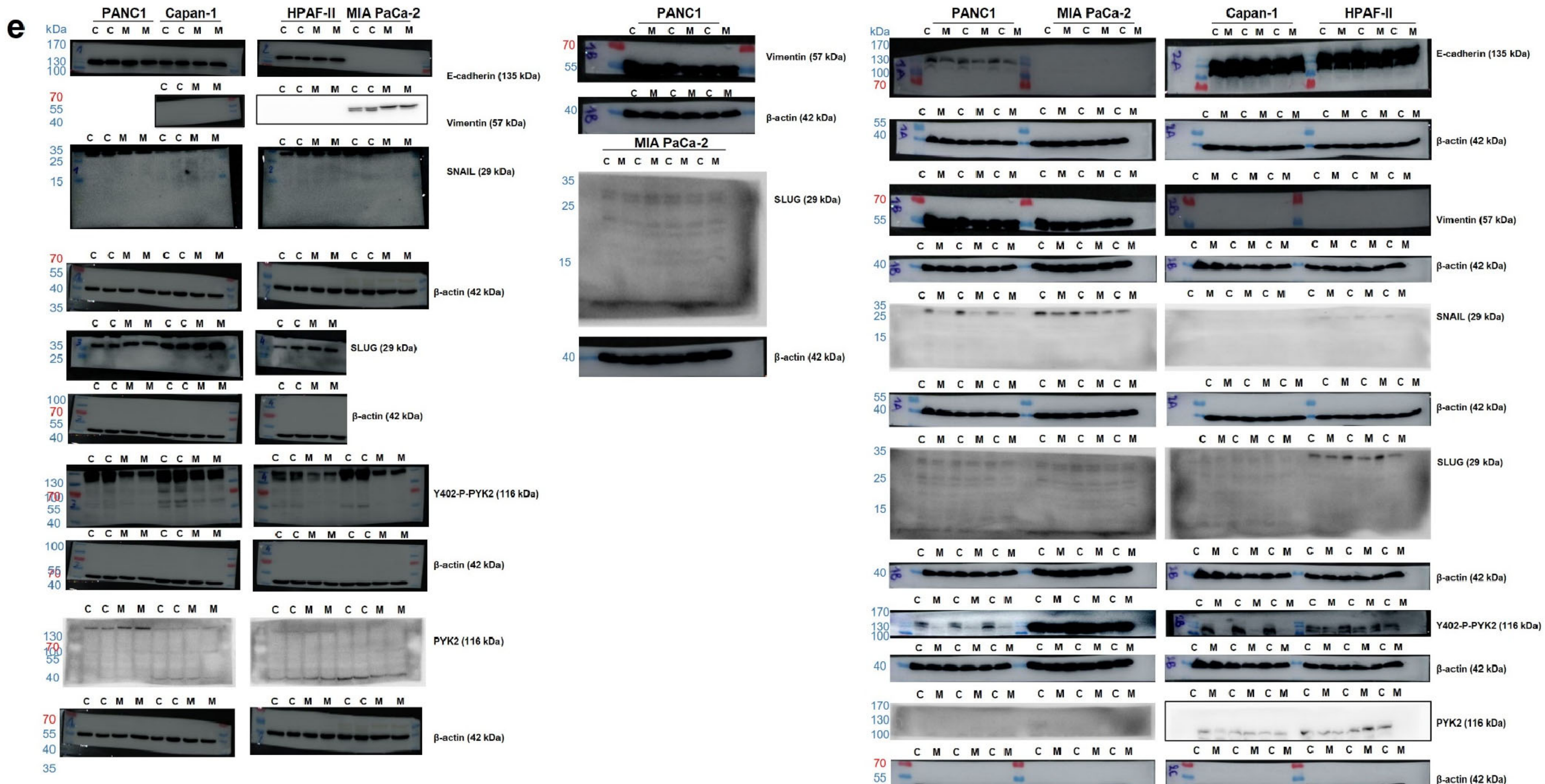

e
PANC1
Capan-1
HPAF-II
MIA PaCa-2
E-cadherin (135 kDa)
Vimentin (57 kDa)
SNAIL (29 kDa)
SLUG (29 kDa)
Y402-P-PYK2 (116 kDa)
PYK2 (116 kDa)
β-actin (42 kDa)
kDa

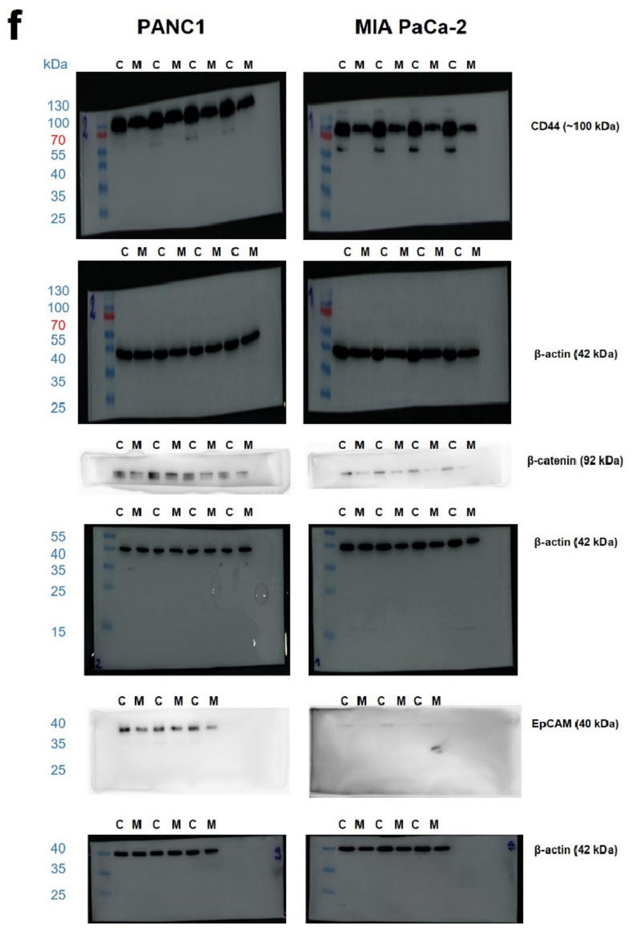
f
PANC1
MIA PaCa-2
kDa
C M C M C M C M
130
100
70
55
40
35
25
CD44 (~100 kDa)
β-actin (42 kDa)
β-catenin (92 kDa)
15
EpCAM (40 kDa)

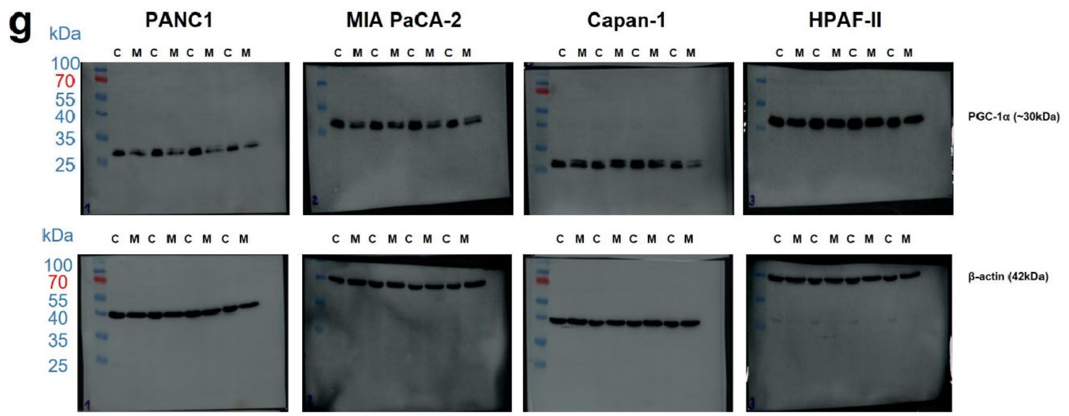
g
PANC1
MIA PaCA-2
Capan-1
HPAF-II
kDa
C M C M C M C M
100
70
55
40
35
25
PGC-1α (~30kDa)
β-actin (42kDa)

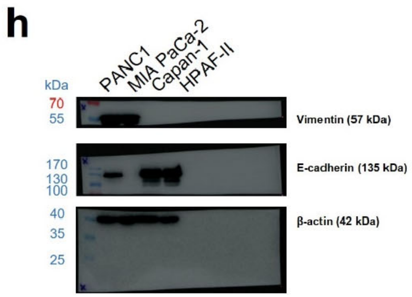
h
PANC1
MIA PaCa-2
Capan-1
HPAF-II
kDa
70
55
Vimentin (57 kDa)
170
130
100
E-cadherin (135 kDa)
40
35
25
β-actin (42 kDa)

Figure S7. Uncropped western blot images. Shown are whole western blot images corresponding to (**a**) **Figure 3c**; (**b**) **Figure 4a**; (**c**) **Figure 4b**; (**d**) **Figure 4d**; (**e**) **Figure 5b**; (**f**) **Figure 6d**; (**g**) **Figure S3a**; and (**h**) **Figure S5a.** Note that, in order to be able to show molecular ladders, the membranes are shown at the longest exposure time (often overexposed), which does not always correspond to the acquisition time on an ECL imager 600. Abbreviations: C, control; M, MitoQ.
